# Induction of the *cydAB* Operon Encoding the *bd* Quinol Oxidase Under Respiration-Inhibitory Conditions by the Major cAMP Receptor Protein MSMEG_6189 in *Mycobacterium smegmatis*

**DOI:** 10.3389/fmicb.2020.608624

**Published:** 2020-11-30

**Authors:** Eon-Min Ko, Jeong-Il Oh

**Affiliations:** Department of Integrated Biological Science, Pusan National University, Busan, South Korea

**Keywords:** *aa3* cytochrome *c* oxidase, cAMP, Crp, electron transport chain, *Mycobacterium*, regulation of gene expression, respiration

## Abstract

The respiratory electron transport chain (ETC) of *Mycobacterium smegmatis* is terminated with two terminal oxidases, the *aa*_3_ cytochrome *c* oxidase and the cytochrome *bd* quinol oxidase. The *bd* quinol oxidase with a higher binding affinity for O_2_ than the *aa*_3_ oxidase is known to play an important role in aerobic respiration under oxygen-limiting conditions. Using relevant *crp1* (*MSMEG_6189*) and *crp2* (*MSMEG_0539*) mutant strains of *M. smegmatis*, we demonstrated that Crp1 plays a predominant role in induction of the *cydAB* operon under ETC-inhibitory conditions. Two Crp-binding sequences were identified upstream of the *cydA* gene, both of which are necessary for induction of *cydAB* expression under ETC-inhibitory conditions. The intracellular level of cAMP in *M. smegmatis* was found to be increased under ETC-inhibitory conditions. The *crp2* gene was found to be negatively regulated by Crp1 and Crp2, which appears to lead to significantly low cellular abundance of Crp2 relative to Crp1 in *M. smegmatis*. Our RNA sequencing analyses suggest that in addition to the SigF partner switching system, Crp1 is involved in induction of gene expression in *M. smegmatis* exposed to ETC-inhibitory conditions.

## Introduction

The respiratory electron transport chain (ETC) of mycobacteria consists of the membrane-associated electron carriers (menaquinone/menaquinol and cytochrome *c*) and enzymes that catalyze electron-transfer reactions with the concomitant generation of proton motive force (Cook et al., 2014). The respiratory ETC of *Mycobacterium smegmatis* is terminated with two terminal oxidases like that of *Mycobacterium tuberculosis*, the *aa*_3_ cytochrome *c* oxidase and the cytochrome *bd* quinol oxidase, which catalyze the reduction of O_2_ to water molecules using the electrons from reduced cytochrome *c* and menaquinol, respectively (Kana et al., 2001; Matsoso et al., 2005). The *aa*_3_ cytochrome *c* oxidase, which belongs to the heme-copper superfamily of oxidases (HCOs) and serves as the major oxidase under aerobic conditions, forms a supercomplex with the cytochrome *bc*_1_ complex and cytochrome *c* (Matsoso et al., 2005; Megehee et al., 2006). Although the cytochrome *bd* quinol oxidase is not capable of pumping protons across the membrane during the reduction of O_2_, it has a higher affinity for O_2_ and is much less sensitive to inhibition by cyanide (CN^−^) than the *aa*_3_ oxidase (Puustinen et al., 1991; Cunningham et al., 1997; Kana et al., 2001; Belevich et al., 2005, 2007). It has been also demonstrated that the *bd* quinol oxidase is relatively insensitive to the physiologically relevant respiration-inhibiting molecules nitric oxide (NO) and hydrogen sulfide (H_2_S) that are produced by activated or infected host immune cells and serve as inhibitors for the bacterial and mitochondrial HCOs (Mason et al., 2009; Giuffre et al., 2012; Forte et al., 2016). From these findings, it has been suggested that the *bd* quinol oxidase is involved in adaptation of pathogenic bacteria such as *M. tuberculosis* to hostile environments created by host immunity during the infection process (Giuffre et al., 2012; Forte et al., 2016). Although the *bd* quinol oxidase is not essential to *M. smegmatis* at ambient oxygen tensions, it plays an important role in aerobic respiration under hypoxic conditions, as well as under inhibitory conditions of the *bcc*_1_-*aa*_3_ branch (Kana et al., 2001; Matsoso et al., 2005; Aung et al., 2014; Jeong et al., 2018). The *bd* quinol oxidase of *M. smegmatis* is encoded by the *cydAB* operon (*cydA*: *MSMEG_3233, cydB*: *MSMEG_3232*) (Kana et al., 2001; Aung et al., 2014). Inactivation of the *bcc*_1_-*aa*_3_ branch and hypoxic conditions were shown to result in strong upregulation of *cydAB* expression in *M. smegmatis* (Kana et al., 2001; Matsoso et al., 2005; Aung et al., 2014; Jeong et al., 2018). It was also demonstrated that albeit moderately, expression of *cydA* was induced in *M. tuberculosis* exposed to hypoxia and NO, as well as in *M. tuberculosis, Mycobacterium bovis* BCG, and *Mycobacterium marinum* treated with ETC inhibitors such as Q203, bedaquiline, and clofazimine (Shi et al., 2005; Koul et al., 2014; Boot et al., 2017; Kalia et al., 2019).

cAMP is a critical secondary messenger that controls a wide variety of cellular functions in many organisms. The cAMP receptor protein (Crp) is a transcriptional regulator that controls gene expression by recognizing altered cAMP levels in prokaryotic cells. The genome of *M. smegmatis* has two genes (*crp1*: *MSMEG_6189, crp2*: *MSMEG_0539*) encoding the Crp paralogs that show 78% sequence identity at the amino acid level (Sharma et al., 2014; Aung et al., 2015). Sequence homology and biochemical analyses revealed that Crp1 corresponds to the Crp protein (Rv3676) occurring in *M. tuberculosis* (Bai et al., 2005; Stapleton et al., 2010; Sharma et al., 2014; Aung et al., 2015). On the basis of high sequence similarity between the helix-turn-helix (HTH) motifs of Crp1 and Crp2, together with the results of DNA-binding analyses, it was suggested that both Crp proteins recognize and bind to the same consensus sequence (TGTGA-N_6_-TCACA) (Sharma et al., 2014). However, there are significant differences in amino acids forming the cAMP binding pockets of the two proteins, which accounts for their different biochemical properties such as their binding affinity for cAMP and DNA, as well as cAMP-dependent enhancement of the DNA-binding affinity (Sharma et al., 2014; Aung et al., 2015).

The expression and regulation patterns of the *bd* quinol oxidase genes in diverse bacteria are similar to those in mycobacteria. Expression of the genes is commonly induced under hypoxic or anaerobic conditions (Kana et al., 2001; Borisov et al., 2011; Small et al., 2013; Aung et al., 2014; Jeong et al., 2018; Mascolo and Bald, 2020). The regulation mechanism of the *bd* quinol oxidase genes in response to changes in oxygen availability was well-established for several bacteria. In *Escherichia coli*, expression of the *cydAB* operon is controlled by the ArcBA two-component system (TCS) and Fnr (fumarate and nitrate reduction regulatory protein) in response to changes in oxygen tensions (Cotter et al., 1990, 1997; Fu et al., 1991; Cotter and Gunsalus, 1992; Tseng et al., 1996). In *Streptomyces coelicolor* A3, expression of *cydAB* is regulated by the Rex repressor that exerts transcriptional regulation in response to changes in the cellular NADH/NAD^+^ ratio (Brekasis and Paget, 2003). Using both site-directed mutagenesis of a Crp-binding sequence upstream of *cydA* and Electrophoretic mobility shift analysis (EMSA) with purified Crp2 (MSMEG_0539), expression of the *cydAB* operon in *M. smegmatis* was suggested to be positively regulated by Crp (Aung et al., 2014). However, detailed study has not been reported regarding the regulatory mechanism for the induction of *cydAB* expression by inactivation of the *bcc*_1_-*aa*_3_ pathway and whether two CRP paralogs differentially contribute to the regulation of *cydAB* expression. Using relevant *crp1* and *crp2* mutant strains of *M. smegmatis*, we here report the roles of Crp1 and Crp2 in upregulation of *cydAB* expression under respiration-inhibitory conditions.

## Materials and Methods

### Bacterial Strains, Plasmids, and Culture Conditions

The bacterial strains and plasmids used in this study are listed in Supplementary Table 1. *E. coli* strains were grown in Luria-Bertani (LB) medium at 37°C. *M. smegmatis* strains were grown in 7H9-glucose medium [Middlebrook 7H9 medium (Difico, Sparks, MD) supplemented with 0.2% (w/v) glucose as a carbon source and 0.02% (v/v) Tween 80 as an anticlumping agent] at 37°C. *M. smegmatis* strains were grown aerobically in a 500-ml flask filled with 100 ml of 7H9-glucose medium on a gyratory shaker (200 rpm). For treatment of *M. smegmatis* cultures with potassium cyanide (KCN), *M. smegmatis* strains were grown until the optical density at 600 nm (OD_600_) reached 0.45–0.5. Following the addition of KCN to the cultures to a final concentration of 100 μM, the cultures were further grown for 15 min. For treatment of *M. smegmatis* cultures with sodium nitroprusside (SNP; an NO generator) or NaHS (an H_2_S generator), SNP and NaHS were added to the *M. smegmatis* cultures grown to an OD_600_ of 0.45–0.5 to final concentrations of 5 mM and 200 μM, respectively, and the cultures were further grown for 30 min. The SNP-treated cultures were grown under illumination of light (100 W/m^2^). Ampicillin (100 μg/ml for *E. coli*), kanamycin (50 μg/ml for *E. coli* and 15 or 30 μg/ml for *M. smegmatis*), and hygromycin (200 μg/ml for *E. coli* and 25 or 50 μg/ml for *M. smegmatis*) were added to the growth medium when required. The construction of the mutants and plasmids used in this study is described in Supplemental Material.

### DNA Manipulation and Transformation

Standard protocols and manufacturers' instructions were followed for recombinant DNA manipulations (Sambrook and Green, 2012). Transformation of *M. smegmatis* with plasmids was carried out by electroporation as described elsewhere (Snapper et al., 1990). The primers used for PCR are listed in Supplementary Table 2.

### Site-Directed Mutagenesis

To introduce point mutations into the Crp-binding sites (CBS1 and CBS2), PCR-based mutagenesis was performed using the Quick Change site-directed mutagenesis procedure (Stratagene, La Jolla, CA). Synthetic oligonucleotides 31 bases long containing the substituted nucleotides in the middle of their sequences were used to mutagenize the sequences. The primers used for mutagenesis are listed in Supplementary Table 2. Mutations were verified by DNA sequencing.

### Quantitative Real-Time PCR

RNA isolation from *M. smegmatis* strains and cDNA synthesis were performed as described elsewhere (Kim et al., 2010) except for the use of a random hexamer primer (ThermoFisher, Waltham, MA) in place of the gene-specific primers in cDNA synthesis. The contamination of DNA in the isolated RNA was checked by PCR with the primers to be used in quantitative real-time PCR (qRT-PCR). To determine the transcript levels of *cydA, crp2, MSMEG_3680*, and *sigA*, qRT-PCR was performed in a 20-μl mixture containing 5 μl of the template cDNA, 15 pmol of each of two gene-specific primers, 10 μl of TB GreenTM Premix Ex TaqTM (Tli RNase Plus) (Takara, Tokyo, Japan), 0.4 μl of the ROX passive fluorescent dye, and 2.6 μl of distilled water. Thermal cycling was initiated with 1 cycle at 95°C for 2 min, followed by 40 cycles of 95°C for 5 s and 64°C for 30 s. The *sigA* gene encoding the principal sigma factor was used as a reference gene for qRT-PCR to normalize the expression levels of *cydA, crp2*, and *MSMEG_3680* since our RNA sequencing analyses revealed that the *sigA* gene is constitutively expressed at similar levels in the wild-type (WT), Δ*aa*_3_, Δ*crp1*, and Δ*crp2* mutant strains (Supplementary Figure 1). Melting curve analysis was performed for each reaction to examine whether a single PCR product was amplified during qRT-PCR. The primers used for qRT-PCR are listed in Supplementary Table 2.

### β-Galactosidase Assay and Determination of the Protein Concentration

The β-Galactosidase activity was measured spectrophotometrically as described previously (Oh and Kaplan, 1999). The protein concentration was determined using a Bio-Rad protein assay kit (Bio-Rad, Hercules, CA) with bovine serum albumin (BSA) as a standard protein.

### Western Blotting Analysis

Cell-free crude extracts were subjected to SDS-PAGE, and proteins on the gel were transferred to polyvinylidene fluoride membranes (Millipore, Burlington, MA). Western blotting analysis using an anti-2B8 antibody (Biojane, Pyeongtaek-si, South Korea) was performed as described previously (Mouncey and Kaplan, 1998). The anti-2B8 antibody was used at a dilution of 1:20,000. To detect GroEL, a mouse monoclonal antibody against Hsp65 (Santa Cruz Biotechnology, Dallas, TX; sc58170) was used at a 1:2,000 dilution. Alkaline phosphatase-conjugated anti-mouse IgG produced in rabbit (Sigma, St. Louis, MO; A4312) was used at a 1:10,000 dilution for the detection of the primary antibodies.

### Protein Purification

C-terminally His_6_-tagged Crp1 was expressed in the *E. coli* BL21 (DE3) strain harboring pT7-7crp1. The *E. coli* strain was cultivated aerobically to an OD_600_ of 0.4–0.6 at 37°C in LB medium containing 100 μg/ml ampicillin. Expression of the *crp1* gene was induced by the addition of isopropyl-β-D-thiogalactopyranoside (IPTG) to the cultures to a final concentration of 0.5 mM, and then cells were further grown for 4 h at 30°C. Cells were harvested from 300 ml cultures and resuspended in 10 ml of buffer A [20 mM Tris-HCl (pH 8.0) and 200 mM NaCl] containing DNase I (10 U/ml) and 10 mM MgCl_2_. The resuspended cells were disrupted twice using a French pressure cell, and cell-free crude extracts were obtained by centrifugation twice at 20,000 × *g* for 15 min. The crude extracts were loaded into a column packed with 500 μl of the 80% (v/v) slurry of Ni-Sepharose high-performance resin (GE Healthcare, Piscataway, NJ). The resin was washed with 40 bed volumes of buffer A containing 5 mM imidazole and washed further with 20 bed volumes of buffer A containing 60 mM imidazole. His_6_-tagged Crp1 was eluted from the resin with 6 bed volumes of buffer A containing 250 mM imidazole. The eluted protein was desalted using a PD-10 desalting column (GE Healthcare) equilibrated with appropriate buffer. The purity of Crp1 was checked by SDS-PAGE (Supplementary Figure 2).

### EMSA

A 99-bp DNA fragment containing the upstream region of *cydA* and an 80-bp control DNA fragment without the Crp-binding site were used in EMSA. The 99-bp DNA fragment was amplified by PCR using pBSIIcydA as a template and the primers F_cydAEMSA and R_cydAEMSA. The 80-bp control DNA fragment was generated by PCR using pUC19 as a template and the primers F_80_EMSA and R_80_EMSA. Purified Crp1 protein was incubated with 70 fmol of the DNA fragments containing the *cydA* upstream region and 100 fmol of the control DNA fragments in binding buffer [20 mM Tris-HCl (pH 8.0), 100 mM NaCl, 2.5 mM MgCl_2_, 1 mM EDTA, 1 mM dithiothreitol (DTT), 50 μg/ml BSA and 10% (v/v) glycerol] in a reaction volume of 10 μl for 20 min at 25°C. After addition of 2 μl of 6x loading buffer (0.25% (w/v) bromophenol blue, 0.25% (w/v) xylene cyanol and 40% (w/v) sucrose), the mixtures were subjected to non-denaturing PAGE [8% (w/v) acrylamide] using 0.5x TBE buffer (41.5 mM Tris-borate and 0.5 mM EDTA, pH8.3) at 70 V for 2 h 20 min at 4°C. The gels were stained with SYBR Green staining solution for 1 h.

### DNase I Footprinting Analysis

DNase I Footprinting was carried out using fluorescence (TAMRA)-labeled DNA fragments and purified Crp1. TAMRA-labeled DNA fragments (271 bp) containing the *cydA* upstream region were generated by PCR using the primers (F_TAMRA_pUC19 and F_cydAFootR) and pUC19cydAFootR as a template. The PCR products were purified after agarose gel electrophoresis. DNA binding reaction mixtures were composed of 5 pmol of labeled DNA probes, purified Crp1 (0.15, 0.3, or 0.6 μM), 20 mM Tris-HCl (pH 8.0), 0.2 mM MgCl_2_, 2.1 mM KCl, 0.04 mM DTT, and 11.1% (v/v) glycerol in a final volume of 190 μl. When necessary, the Crp1 protein was mixed with 200 μM cAMP, and the mixture was incubated for 10 min at 25°C prior to binding reactions for 10 min at 25°C. DNase I (Takara) was diluted in buffer containing 20 mM Tris-HCl (pH 8.0), 50 mM NaCl, 1 mM DTT, and 10% (v/v) glycerol to reach a final concentration of 0.675 mU/μl. DNase I digestion was initiated with the addition of 10 μl of diluted DNase I to the binding reaction mixtures, conducted for 1 min at 25°C, and stopped with the addition of 400 μl of stop solution [20 mM Tris-HCl (pH 8.0) and 40 mM EDTA]. DNA was purified by phenol/chloroform/isoamyl alcohol (25:24:1) extraction and isopropyl alcohol precipitation. The pellets were dissolved in TE buffer [10 mM Tris-HCl (pH 8.0) and 1 mM EDTA]. After the addition of loading buffer [95% deionized formamide, 0.025% (w/v) bromophenol blue, 0.025% (w/v) xylene cyanol FF, and 5 mM EDTA (pH 8.0)], the samples were analyzed by electrophoresis on 6% (w/v) denaturing polyacrylamide gels with 7 M urea in 0.8x Tris-taurine-EDTA (TTE) buffer using an ABI PRISM 377 DNA sequencer (Applied Biosystems, Foster City, CA). Reference sequencing was performed by using a Thermo sequenase dye primer manual cycle sequencing kit (ThermoFisher) with the primer F_TAMRA_pUC19 and the template plasmid pUC19cydAFootR.

### Determination of the Intracellular cAMP Concentration

*M. smegmatis* cells corresponding to 1 ml of cultures at OD_600_ of 0.4 were harvested. Cell pellets were resuspended in 1 ml of 0.1 M HCl and then incubated for 10 min. Cells were disrupted once by using a Fastprep 120 beadbeater (ThermoFisher) at 5.0 m/s for 45 s. Cell-free supernatants were obtained by centrifugation at 20,000 × *g* for 10 min. The concentration of cAMP in the prepared supernatants was determined by using a DetectX Direct Cyclic AMP Enzyme Immunoassay kit (Arbor Assays, Ann Arbor, MI) and a microplate reader (Bio-Rad) following the manufacture's instruction.

### RNA Sequencing and Gene Expression Profiling

Three biological replicate cultures of the WT and Δ*crp1* strains were grown aerobically to an OD_600_ of 2.0–2.1 (late exponential phase). Total RNA of each culture was isolated as described previously (Kim et al., 2010). rRNA was removed from the isolated total RNA using a Ribo-Zero rRNA Removal Kit (Bacteria) (Illumina, San Diego, CA). The RNA sequencing libraries were created using a TruSeq RNA Sample Prep Kit v2 (Illumina) with the standard low-throughput protocol. Sequencing of the six libraries was conducted on an Illumina HiSeq 4000 platform at Macrogen Inc. (Seoul, South Korea) using the Hiseq 3000–4000 sequencing protocol and TruSeq 3000–4000 SBS Kit v3 reagent (Illumina). Paired-end reads (101 bp) were then mapped to the reference genome sequence of *M. smegmatis* mc^2^155 (GCF_000015005.1_ASM1500v1) with the program Bowtie 1.1.2 using default settings. Summarized statistics of RNA sequencing alignment are listed in Supplementary Table 3. The differentially expressed genes (DEGs) were subsequently identified pair-wise by the edgeR package in R language (Robinson et al., 2010). In this analysis, the genes with *p* < 0.05 and |FC| > 1.5 were regarded as DEGs. The RNA sequencing data have been deposited in NCBI's Gene Expression Omnibus and are accessible through the GEO Series accession number GSE158137.

## Results

### Induction of *cydA* Expression Under Respiration-Inhibitory Conditions Is Independent of SigF

**Figure 1:**
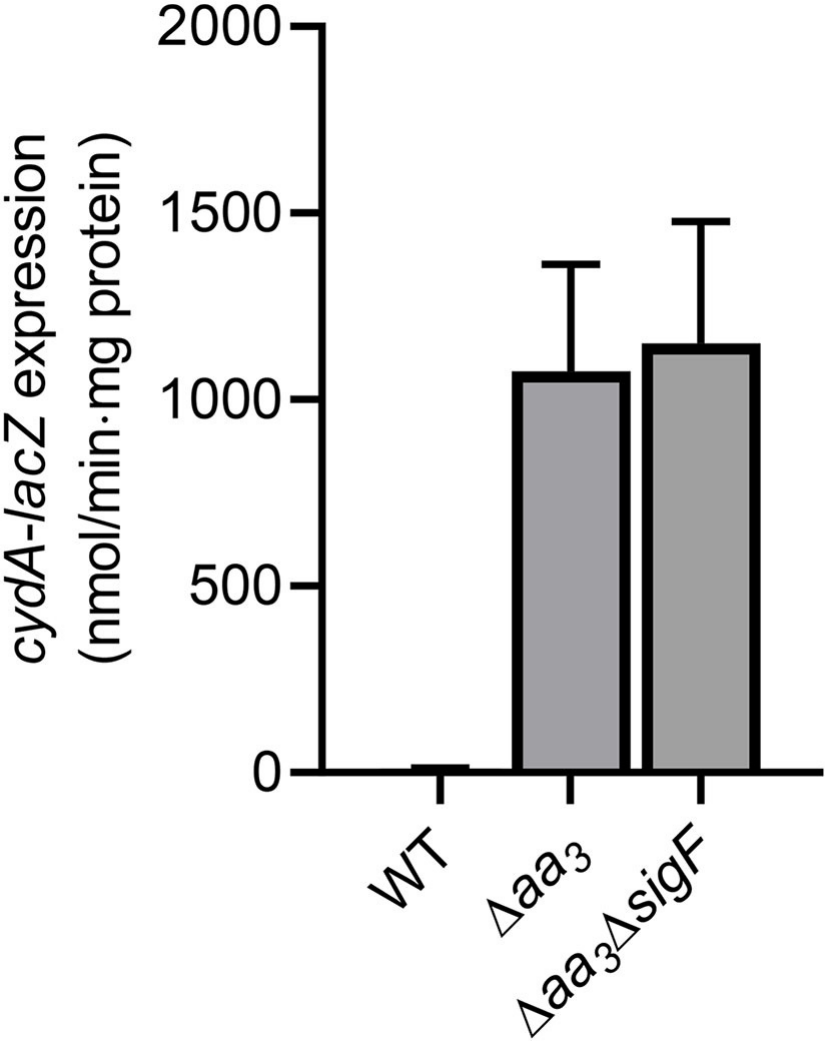
Expression of the *cydAB* operon in the WT, Δ*aa*_3_, and Δ*aa*_3_Δ*sigF* mutant strains of *M. smegmatis*. The WT, Δ*aa*_3_, and Δ*aa*_3_Δ*sigF* mutant strains of *M. smegmatis* harboring the *cydA::lacZ* translational fusion plasmid pNCIIcydA were grown aerobically to an OD_600_ of 0.45–0.5 in 7H9-glucose medium. Cell crude extracts were used to determined β-galactosidase activity. All values provided are the averages of the results from three biological replicates. The error bars indicate the standard deviations.

Under aerobic culture conditions, the inactivation of the *aa*_3_ cytochrome *c* oxidase in *M. smegmatis* by mutation was previously shown to lead to an overall reduction in the respiration rate by ~50% and a significant increase in expression of the *cydAB* operon encoding the *bd* quinol oxidase (Jeong et al., 2018). Furthermore, we found that the genes, which belong to the SigF (an alternative sigma factor) regulon, are strongly upregulated in a mutant strain of *M. smegmatis* lacking the *aa*_3_ oxidase (Oh et al., 2020). To examine whether the induction of *cydAB* expression in the Δ*aa*_3_ mutant with a deletion in *ctaC* encoding subunit III of the *aa*_3_ cytochrome *c* oxidase is a result of SigF activation, we determined expression of *cydA* in the WT, Δ*aa*_3_ mutant, and Δ*aa*_3_Δ*sigF* double mutant strains using the strains harboring the *cydA*::*lacZ* translational fusion plasmid pNCIIcydA. In good agreement with the previous report (Matsoso et al., 2005; Jeong et al., 2018), the expression level of *cydA* was increased in the Δ*aa*_3_ mutant by ~107-fold relative to the WT strain (Figure 1). The expression level of *cydA* was not decreased in the Δ*aa*_3_Δ*sigF* mutant compared to the Δ*aa*_3_ mutant, indicating that the *cydAB* operon does not belong to the SigF regulon, and that the strong upregulation of the *cydAB* operon under respiration-inhibitory conditions is not caused by the activation of SigF.

### MSMEG_6189 Is the Major Crp in *M. smegmatis*

**Figure 2:**
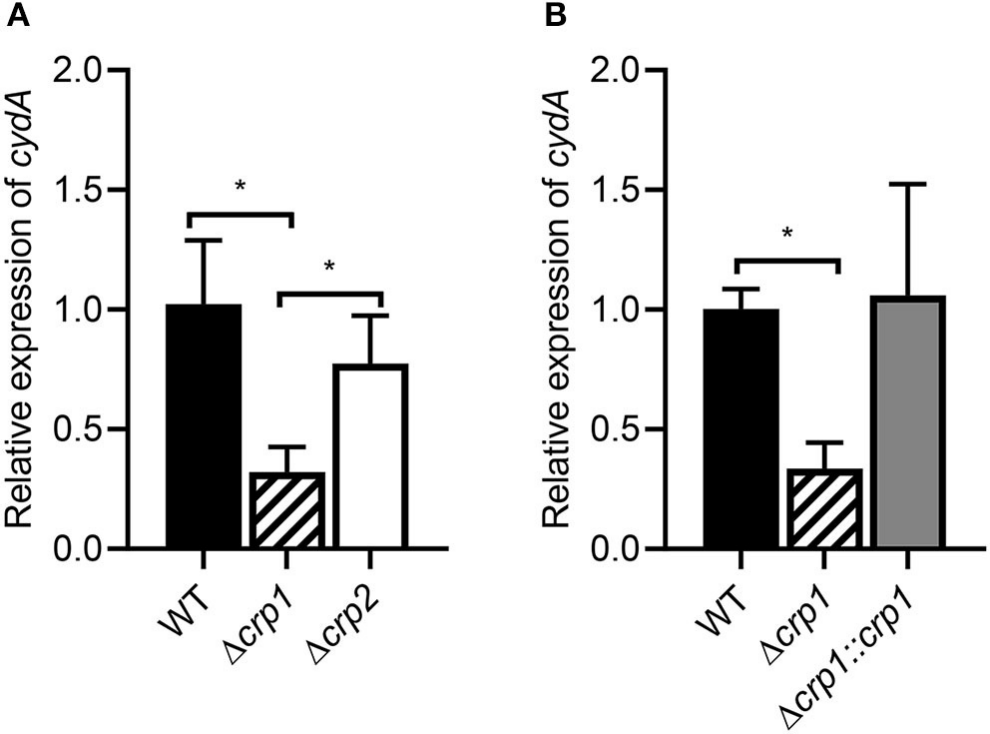
Expression of the *cydAB* operon in the WT, Δ*crp1*, and Δ*crp2* mutant strains of *M. smegmatis*. **(A)** The relative transcript level of *cydA* in the WT, Δ*crp1*, and Δ*crp2* mutant strains. **(B)** Complementation of the Δ*crp1* mutant. For complementation of the Δ*crp1* mutant, pMV306crp (a pMV306-derived plasmid carrying the intact *crp1* gene and its own promoter) was introduced into the mutant. As control strains, the WT and Δ*crp1* strains with the empty vector pMV306 were used in the experiment. All the strains were grown aerobically to an OD_600_ of 0.45–0.5 and treated with 100 μM KCN for 15 min. The expression level of *cydA* was quantitatively determined by qRT-PCR and normalized to *sigA* (the gene for the principal sigma factor) expression. The expression level of *cydA* in the KCN-treated WT strain is set at 1, and the relative values are expressed for the mutant strains. All values provide are the average of the results from three biological replicates. The error bars indicate the standard deviations. Statistical significance was determined by two-tailed Student's *t*-test. *p < 0.05.

Previously it has been reported that Crp is involved in the positive regulation and hypoxic induction of the *cydAB* operon in *M. smegmatis* (Aung et al., 2014). However, it remained unanswered whether two Crp paralogs (Crp1: MSMEG_6189, Crp2: MSMEG_0539) play a distinct role in induction of the *cydAB* operon under respiration-inhibitory conditions. To examine the involvement of Crp1 and Crp2 in the regulation of *cydA* expression, we determined the expression level of *cydA* in the Δ*crp1* and Δ*crp2* mutant strains. Since we failed to obtain a Δ*aa*_3_Δ*crp1* double mutant strain, treatment of *M. smegmatis* cultures with KCN, an inhibitor of *aa*_3_ cytochrome *c* oxidase, was applied to mimic the Δ*aa*_3_ mutant conditions. Effects of KCN treatment on *cydA* expression were quantitatively determined by qRT-PCR in the WT, Δ*crp1*, and Δ*crp2* mutant strains that were grown aerobically. We could not use the *cydA*::*lacZ* transcriptional fusion pNCIIcydA to determine the expression level of *cydA* in the *M. smegmatis* strains treated with KCN, since the addition of KCN interfered with expression or assay of β-galactosidase for unknown reasons. The treatment of the WT strain with 100 μM KCN led to induction of *cydA* expression by 388-fold compared to the untreated WT control strain (data not shown). When the aerobically grown WT, Δ*crp1*, and Δ*crp2* mutant strains were treated with KCN, the Δ*crp1* mutant showed only 30% of *cydA* expression observed in the WT strain, while the expression level of *cydA* was only slightly reduced in the Δ*crp2* mutant relative to the WT strain (Figure 2A). This result indicates that Crp1 plays a predominant role in induction of *cydA* expression under respiration-inhibitory conditions. The ectopic expression of the intact *crp1* gene in the Δ*crp1* mutant using pMV306crp restored the expression level of *cydA* to that in the WT strain with the empty integration vector pMV306 (Figure 2B), confirming that the reduction of *cydA* expression in the Δ*crp1* mutant is the result of *crp1* inactivation. To confirm the Crp1-mediated induction of *cydA* expression by inactivation of the *aa*_3_ cytochrome *c* oxidase, effects of NO and H_2_S, which are the physiologically relevant inhibitors of the *aa*_3_ oxidase, on *cydA* expression were assessed in the WT and Δ*crp1* mutant strains (Supplementary Figure 3). As in the Δ*aa*_3_ mutant and the WT strain treated with KCN, expression of *cydA* was significantly (325-fold) increased in the WT strain treated with 5 mM SNP (NO generator) relative to that in the SNP-untreated control WT strain. Induction of *cydA* expression was significantly compromised in the SNP-treated Δ*crp1* mutant compared to the WT strain treated with SNP. Similarly, expression of *cydA* was increased by 114-fold in the WT strain treated with 200 μM NaHS (H_2_S generator) relative to that in the NaHS-untreated control WT strain. The expression level of *cydA* in the NaHS-treated Δ*crp1* mutant was shown to amount to ~47% of that observed for the NaHS-treated WT strain. Using KCN, we examined the aerobic growth of the WT, Δ*crp1*, and Δ*crp2* mutant strains when the *aa*_3_ oxidase is inhibited (Supplementary Figure 4). The WT, Δ*crp1*, and Δ*crp2* strains grew at the similar rate in 7H9-glucose medium in the absence of KCN. In contrast, the growth of the Δ*crp1* mutant was severely compromised in the presence of 100 μM KCN, and that of the Δ*crp2* mutant was moderately affected compared to the WT strain. The high susceptibility of Δ*crp1* mutant to SNP regarding inhibition of aerobic growth was also demonstrated previously (Lee et al., 2014). Altogether, these results reinforce that inhibition of the respiratory ETC by inactivation of the *aa*_3_ oxidase leads to Crp1-mediated induction of the *cydAB* operon in *M. smegmatis*.

**Figure 3:**
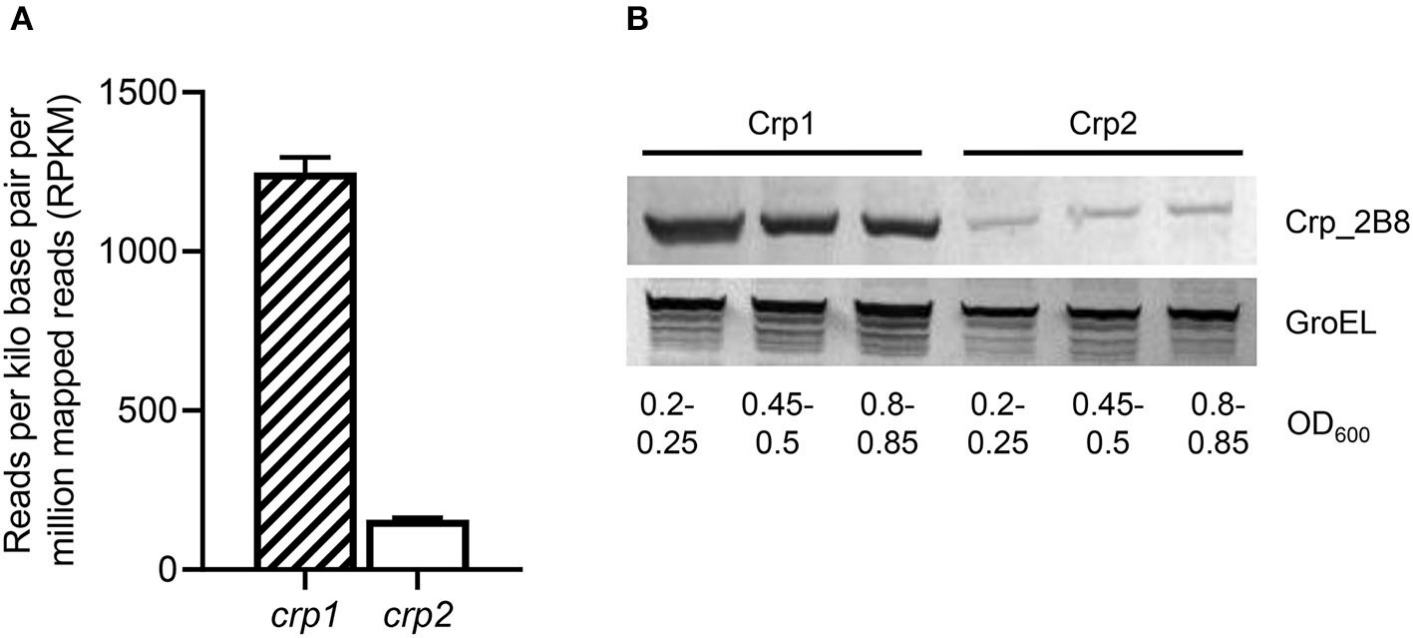
Expression levels of two Crp paralogs in *M. smegmatis*. **(A)** Transcript levels of *crp1* and *crp2* were extrapolated from the RPKM values obtained from RNA sequencing analysis on the WT strain of *M. smegmatis* (Lee et al., 2018). **(B)** Expressed protein levels of Crp1 and Crp2. To determine the protein levels of Crp1 and Crp2 expressed in *M. smegmatis*, the Δ*crp1* and Δ*crp2* mutant strains complemented with pMV306crp1_2B8 and pMV306crp2_2B8, respectively, were grown aerobically to the indicated OD_600_ in 7H9-glucose medium, and their crude extracts (30 μg for detection of Crp1 and Crp2; 5 μg for GroEL detection) were subjected to Western blotting analyses using a 2B8 antibody and an Hsp65 antibody to detect the C-terminally 2B8-tagged Crp and GroEL, respectively. The protein level of GroEL was determined as a loading control.

The expression levels of *crp1* and *crp2* in *M. smegmatis* were extrapolated from the reads per kilo base pair per million mapped reads (RPKM) values obtained from RNA sequencing analysis on the WT strain of *M. smegmatis* that was aerobically grown to an OD_600_ of 0.45–0.5 (Lee et al., 2018). The RPKM values of *crp1* and *crp2* suggested that the transcript level of *crp1* is 8-fold higher than that of *crp2* in the WT strain (Figure 3A). To assess whether the estimated transcript levels of *crp1* and *crp2* correlate with their cellular protein levels, Western blotting analysis was performed using the Δ*crp1* and Δ*crp2* mutant strains expressing the C-terminally 2B8 epitope-tagged Crp1 and Crp2 proteins, respectively. For the construction of the strains, the *crp1* and *crp2* genes with the upstream regions encompassing their own promoters and regulatory sequences were cloned into the integration vector pMV306, and the resulting pMV306crp1_2B8 and pMV306crp2_2B8 plasmids were integrated into the chromosomes of the Δ*crp1* and Δ*crp2* mutant strains, respectively. Western blotting analysis revealed that Crp1 was expressed at much higher levels than Crp2 in *M. smegmatis* grown to various stages of exponential growth phase, while the protein level of GroEL, which was used as a loading control, was relatively constant in both strains grown to various stages of exponential growth phase (Figure 3B). This finding that Crp1 is the predominantly expressed Crp in *M. smegmatis* might explain the dominant role of Crp1 in the positive regulation of the *cydAB* operon.

### Identification of Two Crp-Binding Sites in the Upstream Region of *cydA* and Their Roles in *cydA* Expression

**Figure 4:**
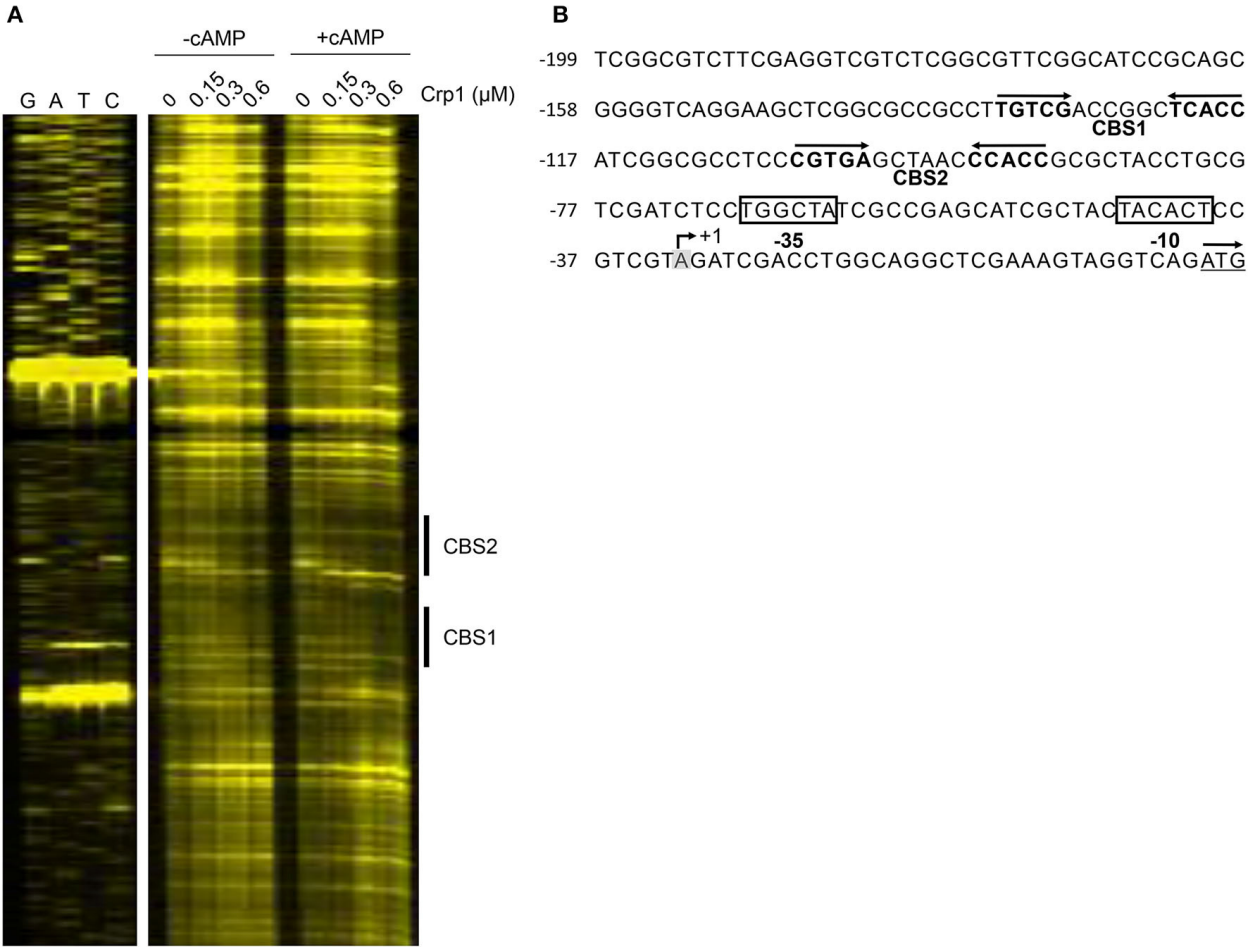
Identification of the Crp-binding sites in the upstream region of *cydA*. **(A)** DNase I Footprinting analysis of the *cydA* upstream region bound by Crp1. The DNA fragments containing the non-coding strands labeled with TAMRA at their 5′ ends were incubated with increasing concentrations of purified Crp1 in the absence or presence of 200 μM cAMP and then subjected to DNase I footprinting reactions. The concentrations of Crp1 used are given above the lanes. The Crp-binding sites (CBS1 and CBS2) are marked by the thick lines on the right. Lanes G, A, T, and C represent the sequence ladders. **(B)** The upstream sequence of the *cydA* gene. The Crp-binding sites (CBS1 and CBS2) are marked by the two head-facing arrows above the sequence. The nucleotide reported to be the TSP (+1) of *cydA* is shaded in gray, and the putative −10 and −35 elements extrapolated from +1 are boxed (Aung et al., 2014). The start codon of *cydA* is underlined and the arrow above it indicates the transcriptional direction. The numbers on the left of the sequences indicate the positions of the leftmost nucleotides relative to the *cydA* gene. The concentration of Crp1 refers to that of the Crp1 monomer.

**Figure 5:**
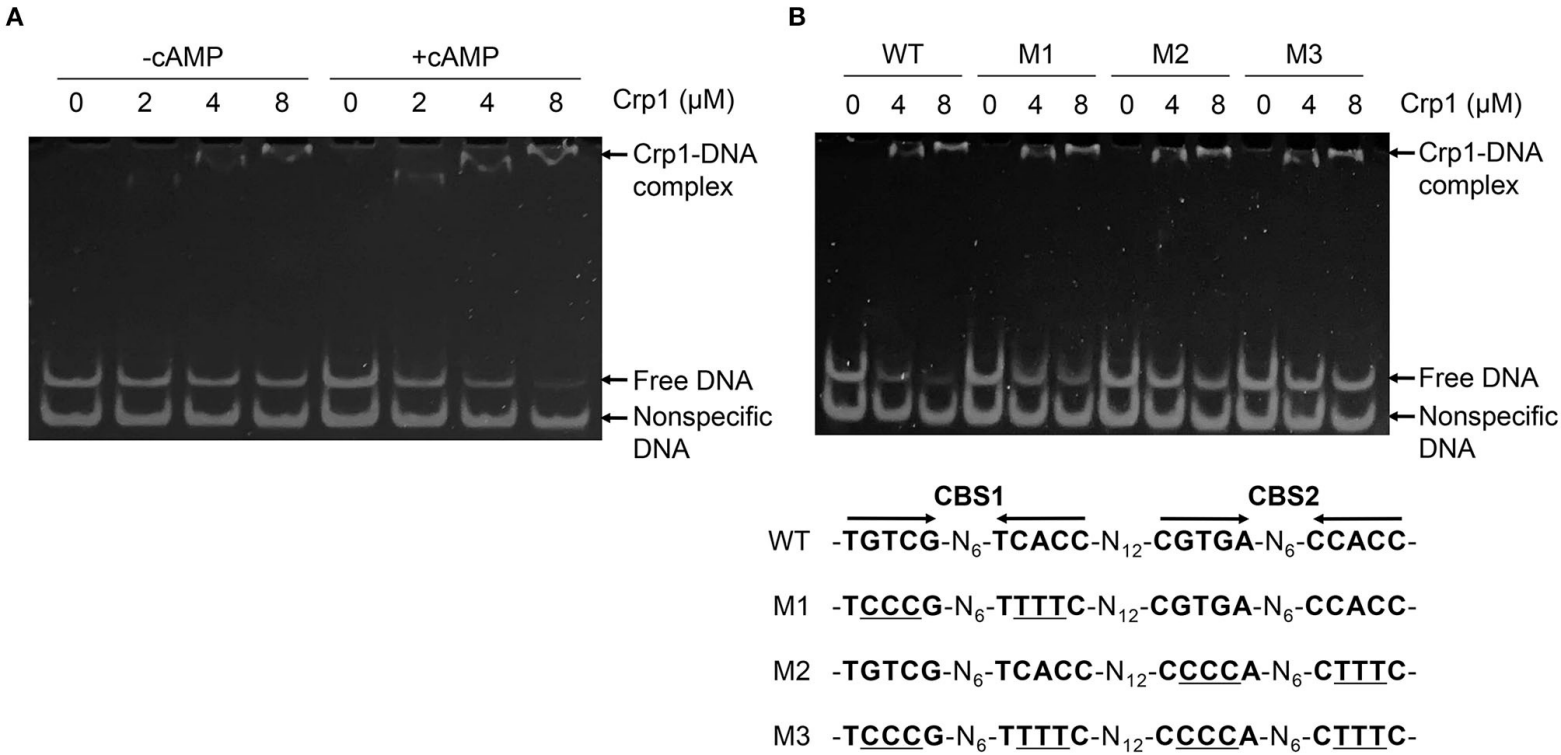
Binding of Crp1 to the *cydA* control region. **(A)** EMSA showing the effect of cAMP on the binding of Crp1 to the upstream region of *cydA*. The mixtures of 99-bp DNA fragments (70 fmol) containing two Crp-binding sites (CBS1 and CBS2) upstream of *cydA* and 80-bp non-specific DNA fragments without the Crp-binding site (100 fmol) were incubated with increasing concentrations of purified Crp1 in the presence and absence of 200 μM cAMP. **(B)** 99-bp DNA fragments (70 fmol) containing either WT or mutated Crp-binding sites (M1, M2, and M3), as well as 80-bp non-specific DNA fragments without the Crp-binding site (100 fmol), were mixed with increasing concentrations of purified Crp1 in the presence of 200 μM cAMP. The Crp1-DNA reaction mixtures were subjected to native PAGE. The concentrations of Crp1 used in EMSA are given above the lanes. The concentration of Crp1 refers to that of the Crp1 monomer.

To identify the Crp-binding sequence(s) in the upstream region of *cydA*, DNase I footprinting analysis was performed with purified Crp1 and 271-bp TAMRA-labeled DNA fragments containing the *cydA* promoter region. Since Crp1 was shown to play a predominant role in the regulation of the *cydAB* operon and both Crp paralogs were suggested to bind to the same DNA sequence (Sharma et al., 2014), we used only Crp1 for DNA-binding analyses. As shown in Figure 4A, binding of Crp1 protected DNA from DNase I cleavage at positions between −52 and −102 with regard to the transcription start point (TSP) of *cydA*. The protected region contains two Crp-binding sequences (CBS1 and CBS2) that are similar to the known Crp-binding consensus sequence (TGTGA-N_6_-TCACA) (Figure 4B). CBS1 is located at positions between −86 and −101 relative to the TSP, and CBS2 was located between −58 and −73. The addition of cAMP to the reaction mixtures resulted in wider and more clearly protected windows for both CBS1 and CBS2, indicating that cAMP enhances the binding of Crp1 to both Crp-binding sites. At low Crp1 concentrations, CBS2 was protected better than CBS1 in the presence and absence of cAMP, indicating that Crp1 binds better to CBS2 than it does to CBS1. To confirm the result of DNase I footprinting, EMSAs were performed with purified Crp1 and 99-bp DNA fragments encompassing the *cydA* upstream sequence (Figure 5). An 80-bp DNA fragment without the Crp-binding sequence (non-specific DNA) was used as a negative control DNA. The binding ability of Crp1 for the DNA fragments was estimated from the band intensity of the unretarded free DNA. As shown in Figure 5A, the binding of Crp1 to the *cydA* regulatory region was enhanced in the presence of 200 μM cAMP, which is consistent with the DNase I footprinting result. In order to determine to what extent mutations of each Crp-binding site affect the binding of Crp1 to the *cydA* regulatory region, we performed EMSAs using purified Crp1 and 99-bp DNA fragments containing the WT or mutated Crp-binding sites (M1, M2, and M3) in the presence of cAMP. As shown in Figure 5B, the M1 and M2 DNA fragments containing mutations within CBS1 and CBS2, respectively, were retarded by Crp1 to a lesser extent than the WT DNA fragment as judged by the levels of free DNA. Especially the M2 mutation significantly affected the binding of Crp1 to the *cydA* regulatory region. Mutations of both CBS1 and CBS2 virtually abolished the binding of Crp1 to the M3 DNA fragment. Taken together, the EMSA and DNase I footprinting results indicate that Crp1 can bind to both CBS1 and CBS2 with a higher binding affinity for CBS2.

**Figure 6:**
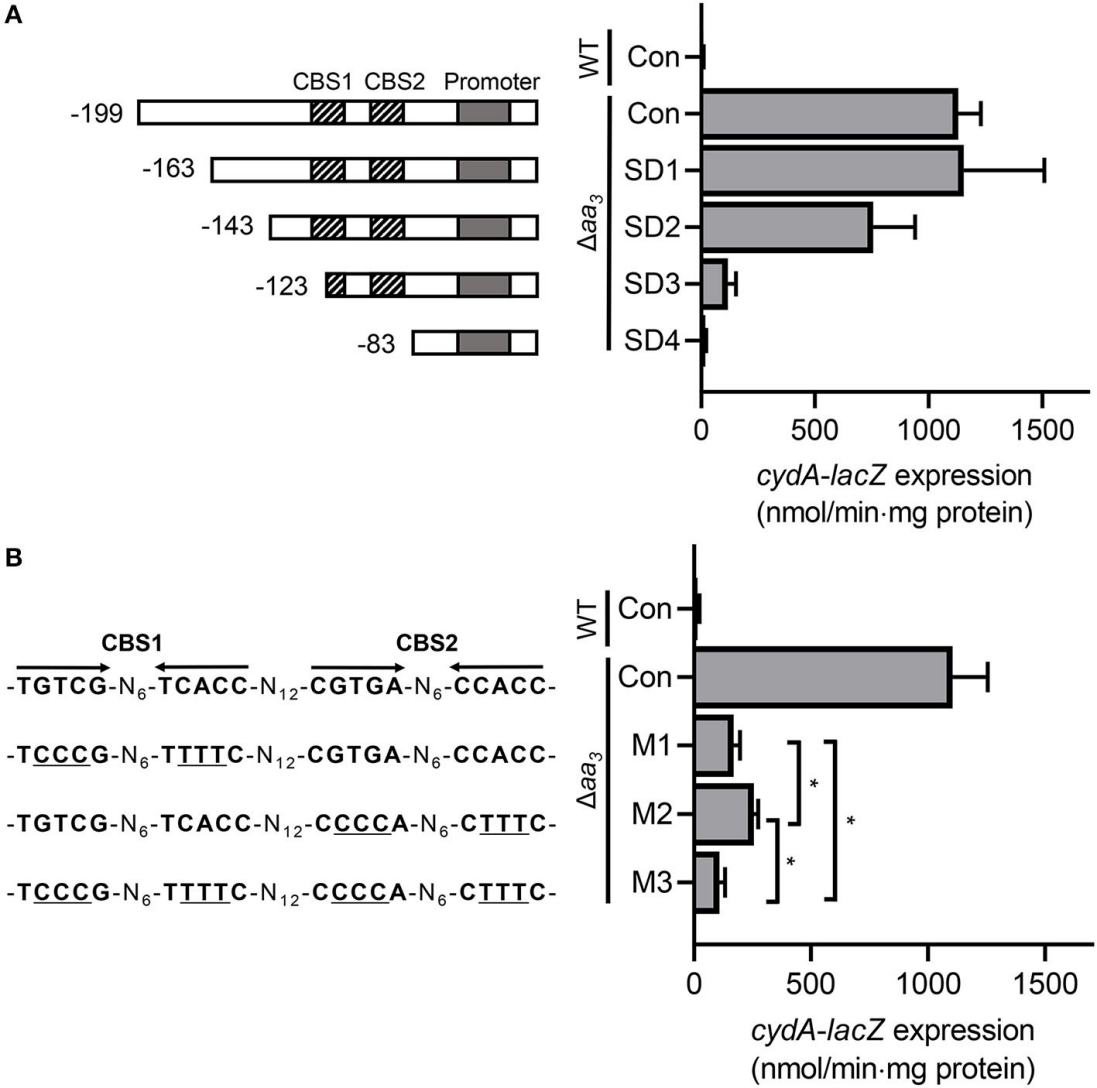
Effects of deletions and mutations in the Crp-binding sites (CBS1 and CBS2) on expression of the *cydAB* operon. **(A)** The *cydA* promoter activity was determined using the pNCIIcydA-derived *cydA::lacZ* translational fusions containing serial deletions of the *cydA* upstream region (pNCIISD1, pNCIISD2, pNCIISD3, and pNCIISD4). **(B)** The *cydA* promoter activity was determined using several pNCIIcydA derivatives containing mutations in the Crp-binding sites (pNCIIM1, pNCIIM2, and pNCIIM3). The mutations in the Crp-binding sites of the *cydA*::*lacZ* translational fusions are presented on the left. The mutations within CBS1 and CBS2 are indicated by underlines. The WT and Δ*aa*_3_ mutant strains of *M. smegmatis* harboring the *cydA::lacZ* translational fusion plasmids were grown aerobically to an OD_600_ of 0.45–0.5 in 7H9-glucose medium. Cell crude extracts were used to determined β-galactosidase activity. All values provided are the averages of the results from three biological replicates. The error bars indicate the standard deviations. Statistical significance was determined by two-tailed Student's *t*-test. *p < 0.05. Con, pNCIIcydA; SD1-SD4, pNCIISD1- pNCIISD4; M1-M3, pNCIIM1-pNCIIM3.

To investigate the role of CBS1 and CBS2 in induction of *cydA* expression under respiration-inhibitory conditions, a series of *cydA*::*lacZ* translational fusions with 5'-serial deletions of the *cydA* upstream region [pNCIISD1 (SD1), pNCIISD2 (SD2), pNCIISD3 (SD3), and pNCIISD4 (SD4)] were used to determine expression of *cydA* in the Δ*aa*_3_ mutant of *M. smegmatis* (Figure 6A). Consistent with the result presented in Figure 1, expression of *cydA* was strongly induced in the Δ*aa*_3_ mutant carrying pNCIIcydA (Con) relative to the control WT strain with pNCIIcydA. The 5'-deletion up to the position−163 relative to the TSP (SD1) did not affect *cydA* expression in the Δ*aa*_3_ mutant, and the additional 20-bp deletion of the *cydA* upstream region (SD2) led to a ~35% decrease in *cydA* expression in the Δ*aa*_3_ mutant. When the *cydA* upstream region was further deleted to remove the 5′ half of CBS1 (SD3), the induction of *cydA* expression in the Δ*aa*_3_ mutant was almost abolished. The deletion of both CBS1 and CBS2 (SD4) resulted in complete abolishment of *cydA* expression in the Δ*aa*_3_ mutant. To more precisely assess the role of CBS1 and CBS2 in the regulation of *cydA* expression, point mutations were introduced into CBS1, CBS2, or both CBS1 and CBS2 on pNCIIcydA, and the expression level of *cydA* was measured using the Δ*aa*_3_ mutants carrying the corresponding *cydA*::*lacZ* translational fusion plasmids [pNCIIcydA (Con), pNCIIM1 (M1), pNCIIM2 (M2), and pNCIIM3 (M3)] (Figure 6B). As observed for the Δ*aa*_3_ mutants with M1 and M2, the introduction of point mutations into CBS1 (TGTCG-N_6_-TCACC to TCCCG-N_6_-TTTTC) and CBS2 (CGTGA-N_6_-CCACC to CCCCA -N_6_-CTTTC) resulted in only 15 and 23% of *cydA* expression observed in the Δ*aa*_3_ mutant with pNCIIcydA, respectively. The effect of mutations within both CBS1 and CBS2 appeared to be cumulative as judged by the expression level of *cydA* in the Δ*aa*_3_ strain with M3. Altogether, the results presented in Figure 6 suggest that both CBS1 and CBS2 are required for induction of *cydA* expression under respiration-inhibitory conditions. It is noteworthy that expression of *cydA* was still 7.3-fold induced in the Δ*aa*_3_ mutant with M3 compared to the WT strain with pNCIIcydA (Figure 6B). From this finding together with the observed reduction in *cydA* expression from SD2 relative to that from SD1 (Figure 6A), we cannot rule out the possibility that there might be an additional *cis*-acting element within or overlapping the 20-bp sequence between SD1 and SD2, which is implicated in the induction of *cydA* expression in *M. smegmatis* under respiration-inhibitory conditions.

### SigF and Crp1 Are the Major Contributors in Induction of Gene Expression in *M. smegmatis* Under Respiration-Inhibitory Conditions

**Figure 7:**
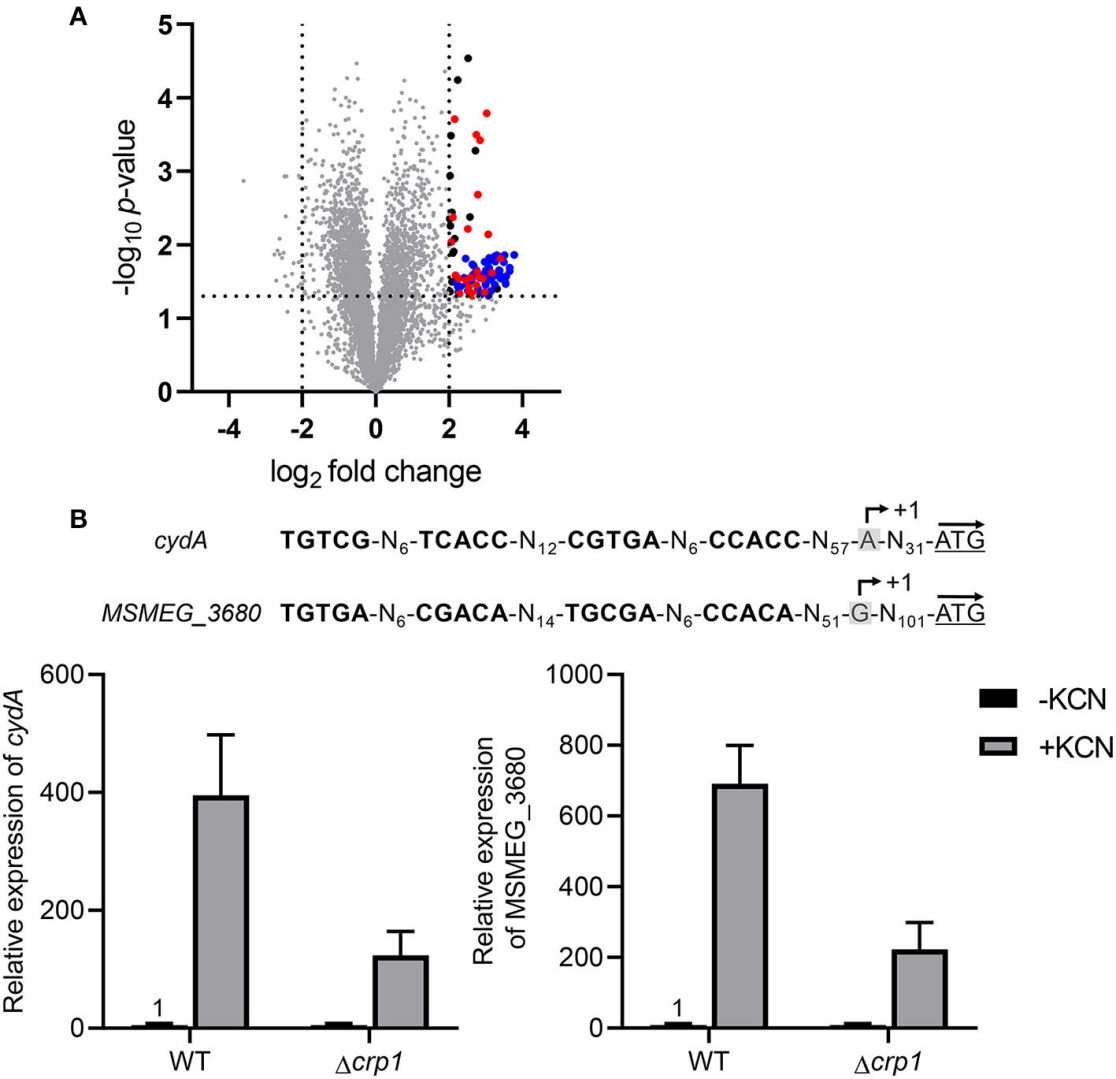
Overlap of the SigF and Crp1 regulons with the genes induced in the Δ*aa*_3_ mutant strain of *M. smegmatis*. **(A)** Volcano plot showing the DEGs in the Δ*aa*_3_ mutant strain relative to the WT strain. RNA sequencing was performed using RNA prepared from three independent replicate cultures of the WT and Δ*aa*_3_ mutant strains grown aerobically in 7H9-glucose medium to an OD_600_ of 0.45–0.5. The x-axis displays the log_2_ fold change of gene expression (log_2_FC) in the Δ*aa*_3_ mutant relative to the WT strain, and the y-axis represents -log_10_ *p*-value. The horizontal dotted line on the graph indicates the border line indicating the *p*-value of 0.05, and the vertical dotted lines indicate the border lines indicating the log_2_FC values of−2 and +2. One hundred and three genes, whose expression is increased by more than log_2_FC ≥ 2 with *p* < 0.05, are depicted by black filled circles. Among the 103 DEGs, the genes belonging to the SigF regulon are denoted by blue filled circles (Singh et al., 2015), and the genes, whose expression is reduced by FC ≥ 1.5 with *p* < 0.05 in the Δ*crp1* mutant strain relative to the WT strain, are indicated by red filled circles. **(B)** The nucleotide sequences and locations of the Crp-binding sites in the upstream regions of *cydA* and *MSMEG_3680* and the expression levels of the genes in the WT and Δ*crp1* mutant strains of *M. smegmatis*. The Crp-binding sites are shown in bold. The previously reported TSPs of *cydA* and *MSMEG_3680* are denoted by +1 (Aung et al., 2014; Martini et al., 2019). The start codons of *cydA* and *MSMEG_3680* are underlined, and the arrows above them indicate the transcriptional direction. The expression levels of *cydA* and *MSMEG_3680* in the WT and Δ*crp1* mutant strains were quantitatively determined by qRT-PCR. The strains were aerobically grown to an OD_600_ of 0.45–0.5 and treated with 100 μM KCN for 15 min (+KCN). As controls, the WT and Δ*crp1* mutant strains grown aerobically without KCN treatment were included in the experiment (-KCN). The expression levels of *cydA* and *MSMEG_3680* determined by qRT-PCR were normalized to that of *sigA*. The expression level of each gene in the KCN-untreated WT strain is set at 1, and the relative values are expressed for the other strains. All values provide are the averages of the results from three independent experiments. The error bars indicate the standard deviations.

Based on the findings that the *cydAB* operon is strongly upregulated in the Δ*aa*_3_ mutant in a Crp-dependent way and does not require SigF for its transcription, we searched for the genes that are regulated in a similar way as the *cydAB* operon to further exemplify the Crp-mediated induction of gene expression under respiration-inhibitory conditions. As shown in Figure 7A, our comparative RNA sequencing analysis on the WT and Δ*aa*_3_ mutant strains revealed 103 DEGs whose expression is increased in the Δ*aa*_3_ mutant by more than 4-fold with a *p* < 0.05 relative to the WT strain. Sixty-one genes among the 103 DEGs were found to belong to the known SigF regulon (Singh et al., 2015). RNA sequencing analysis on the WT and Δ*crp1* mutant strains showed that among the remaining 42 genes, 25 genes was found to be less expressed in the Δ*crp1* mutant by more than 1.5-fold with a *p* < 0.05 relative to the WT strain (Supplementary Table 4). Due to the presence of intact *crp2* in the Δ*crp1* mutant, the less stringent cutoff value (1.5) was applied to select the genes that are positively regulated by Crp1. Among the identified 25 DEGs showing the similar expression patterns as *cydAB* in terms of the strong induction of gene expression in the Δ*aa*_3_ mutant in a SigF-independent way and a decrease in gene expression in the Δ*crp1* mutant, *MSMEG_3680* annotated as a hypothetical protein gene is such a gene that has two putative Crp-binding sites in its upstream region which are arranged similarly as those found upstream of *cydA* (Figure 7B). Using qRT-PCR, the expression pattern of *MSMEG_3680* was verified in the WT and Δ*crp1* mutant strains that were grown aerobically with or without treatment of KCN. In the same way as *cydA* expression, expression of *MSMEG_3680* was strongly induced in the WT strain by treatment of KCN, and induction of its expression by KCN was significantly compromised in the Δ*crp1* mutant. Altogether, the results from RNA sequencing analyses suggest the possibility that Crp1 is likely involved in induction of gene expression in *M. smegmatis* under respiration-inhibitory conditions.

**Figure 8:**
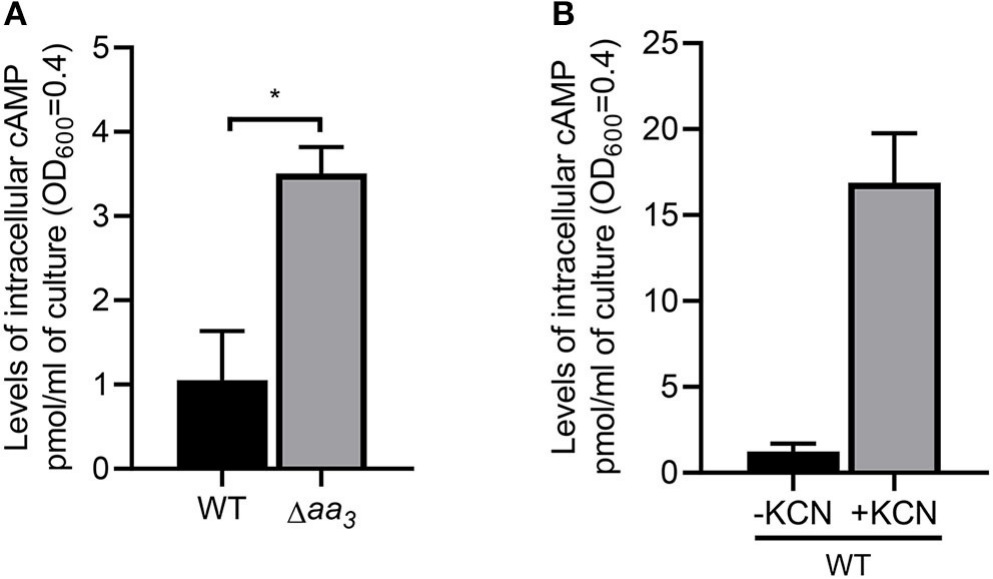
Intracellular levels of cAMP in *M. smegmatis* under respiration-inhibitory conditions. **(A)** Intracellular levels of cAMP in the WT and Δ*aa*_3_ mutant strains of *M. smegmatis*. The WT and Δ*aa*_3_ mutant strains of *M. smegmatis* were grown aerobically to an OD_600_ of 0.45–0.5 in 7H9-glucose medium. **(B)** Effect of KCN treatment on intracellular levels of cAMP in *M. smegmatis*. The WT strain of *M. smegmatis* was grown aerobically to an OD_600_ of 0.45–0.5 in 7H9-glucose medium, and the cultures were treated with 100 μM KCN for 15 min (+KCN). As a control, the WT strain without treatment of KCN was used in the experiment (-KCN). cAMP quantification was performed using a DetectX Direct Cyclic AMP Enzyme Immunoassay kit (Arbor Assays) as described in Materials and Methods. All values provided are the average of the results from three biological replicates. The error bars indicate the standard deviations. Statistical significance was determined by two-tailed Student's *t*-test. *p < 0.05.

Since Crp is a regulatory protein that regulates gene expression in response to changes in cAMP levels, we determined whether the level of cAMP in *M. smegmatis* is changed in response to respiration inhibition. As shown in Figure 8A, the intracellular level of cAMP in the Δ*aa*_3_ mutant grown aerobically was found to be 3.2-fold higher than that detected in the WT strain grown under the same conditions. The level of cAMP was even more (14-fold) increased, when the aerobically grown WT strain of *M. smegmatis* was treated with 100 μM KCN for 15 min relative to the KCN-untreated control WT strain (Figure 8B). The respiratory ETC in the WT strain subjected to a short period (15 min) of KCN treatment is assumed to be more inhibited, at least temporarily before the induced synthesis of the *bd* quinol oxidase, than that in the Δ*aa*_3_ mutant in which expression of the *cydAB* operon is constitutively induced. The higher cAMP level in the KCN-treated WT strain than in the Δ*aa*_3_ mutant likely results from the more severe inhibition of the ETC in the KCN-treated WT strain. These results suggest that inhibition of the respiratory ETC entails an increase in intracellular cAMP levels, which appears to contribute to Crp-mediated induction of *cydA* expression in *M. smegmatis* under respiration-inhibitory conditions.

**Figure 9:**
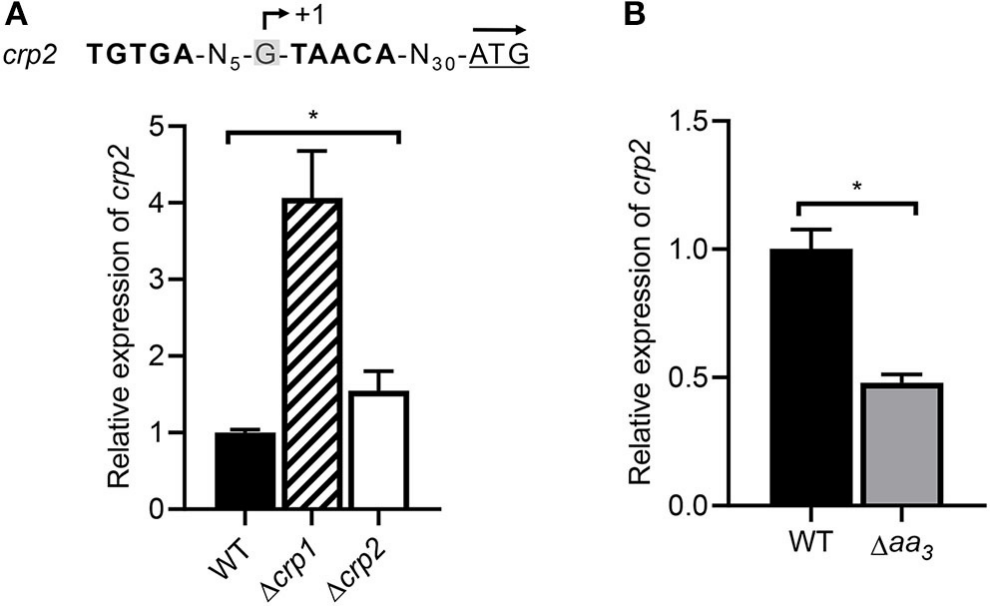
Expression of the *crp2* gene in the WT, Δ*crp1*, Δ*crp2*, and Δ*aa*_3_ mutant strains of *M. smegmatis*. **(A)** Determination of the *crp2* transcript level in the WT, Δ*crp1*, and Δ*crp2* mutant strains by means of qRT-PCR. The positions of the identified Crp-binding site and the TSP (+1) relative to the start codon of *crp2* are shown above the graph (Martini et al., 2019). The start codon of *crp2* is underlined and the arrow above it indicates the transcriptional direction. The strains were grown aerobically to an OD_600_ of 0.45–0.5 and treated with 100 μM KCN for 15 min. The expression level of *crp2* in the KCN-treated WT strain is set at 1, and the relative values are expressed for the mutant strains. **(B)** Determination of the *crp2* transcript level in the WT and Δ*aa*_3_ mutant strains using qRT-PCR. The strains were grown aerobically to an OD_600_ of 0.45–0.5. The expression level of *crp2* in the WT strain is set at 1, and the relative values are expressed for the Δ*aa*_3_ mutant. All values provide are the average of the results from three biological replicates. The error bars indicate the standard deviations. Statistical significance was determined by two-tailed Student's *t*-test. *p < 0.05.

RNA sequencing analysis showed that expression of *crp2* was increased by 3.4-fold in the Δ*crp1* mutant relative to the WT strain, when both strains were grown aerobically (data not shown). We found that a Crp-binding consensus sequence overlaps with the TSP of *crp2* (Figure 9A). Using qRT-PCR, we determined the expression level of *crp2* in the WT, Δ*crp1*, and Δ*crp2* mutant strains after the strains had been treated with KCN (cAMP-increasing conditions). As shown in Figure 9A, expression of *crp2* was increased in the Δ*crp1* and Δ*crp2* mutant strains by 4.1- and 1.5-fold, respectively, when compared to that in the WT strain. This result indicates that *crp2* is under the negative control of Crp1 and Crp2, and that Crp1 predominates in the negative regulation of *crp2* under respiration-inhibitory conditions. We also determined the expression level of *crp2* in the WT and Δ*aa*_3_ mutant strains that were grown aerobically (Figure 9B). The expression level of *crp2* was reduced by 50% in the Δ*aa*_3_ mutant compared to that in the WT strain, suggesting that expression of *crp2* in *M. smegmatis* is even more repressed under respiration-inhibitory conditions probably via the negative regulation by Crp1.

## Discussion

The prokaryotic cytochrome *bd* quinol oxidase is structurally and functionally distinct from the HCOs including the *aa*_3_ cytochrome *c* oxidase (Belevich et al., 2005; Megehee et al., 2006; Borisov et al., 2011). The *bd* quinol oxidase has a higher affinity for O_2_ than HCOs (Puustinen et al., 1991; Belevich et al., 2005, 2007) and has been suggested to possess the additional activities that decompose H_2_O_2_ and peroxynitrite which is a product of the spontaneous reaction of superoxide radical with NO (Lindqvist et al., 2000; Borisov et al., 2004, 2013; Mason et al., 2009; Giuffre et al., 2014). These properties, together with insensitivity of the *bd* quinol oxidase to the physiologically relevant HCO inhibitors NO and H_2_S, make the *bd* quinol oxidase beneficial for bacteria to adapt to and survive in hostile host conditions such as hypoxia and conditions exposed to reactive oxygen species, NO, and H_2_S (Kana et al., 2001; Matsoso et al., 2005; Giuffre et al., 2012; Small et al., 2013; Forte et al., 2016; Rahman et al., 2020; Saini et al., 2020). Accordingly, expression of the *bd* quinol oxidase genes is regulated to be induced under respiration-inhibitory conditions such as hypoxia and in the presence of NO and H_2_S that act as inhibitors of HCOs (Kana et al., 2001; Shi et al., 2005; Giuffre et al., 2012; Small et al., 2013; Aung et al., 2014; Jones-Carson et al., 2016; Jeong et al., 2018). The known regulatory systems, which are involved in the induction of gene expression under hypoxic or anaerobic conditions, generally sense either the molecular oxygen itself or cellular changes caused by the inhibition of the respiratory ETC. The DevSR TCS in *M. smegmatis* senses directly the molecular oxygen through the heme *b* in the DevS histidine kinase and upregulates its target genes under hypoxic and anaerobic conditions (Mayuri et al., 2002; O'Toole et al., 2003; Lee et al., 2008; Kim et al., 2010). The Fnr regulators found in many bacteria also sense O_2_ levels through their O_2_-labile [4Fe-4S] center and regulate gene expression in response to changes in oxygen availability (Kiley and Beinert, 1998). The inhibition of the respiratory ETC by O_2_ depletion or the inactivation (or inhibition) of the ETC components such as the terminal oxidases is expected to entail changes in the redox state of electron carriers to a more reduced state. The ArcB histidine kinase of the ArcBA TCS in *E. coli* and the RegB histidine kinase of the RegBA TCS in *Rhodobacter capsulatus* have been suggested to be activated by increased ubiquinol of the quinol/quinone pool under anaerobic conditions (Georgellis et al., 2001; Malpica et al., 2004; Swem et al., 2006; Bekker et al., 2010; Wu and Bauer, 2010). The Rex repressors in Gram-positive bacteria regulate gene expression in response to changes in the redox poise of the NADH/NAD^+^ pool (Brekasis and Paget, 2003; Schau et al., 2004; Larsson et al., 2005; Gyan et al., 2006). Under respiration-inhibitory conditions, the increased ratio of NADH to NAD^+^ inhibits the DNA-binding activity of Rex via the binding of NADH to the conserved Rossman fold of Rex (Brekasis and Paget, 2003). Since expression of the *cydAB* operon is strongly induced in the Δ*aa*_3_ mutant grown under ambient air conditions, the regulatory system responsible for hypoxic induction of the *cydAB* operon is assumed not to be the regulator that can directly sense the molecular oxygen, but to be the regulatory system that controls gene expression through reflecting the cellular redox state or the functionality of the respiratory ETC. In this respect, the DevSR TCS appears not to be the regulatory system that is responsible for induction of *cydA* in *M. smegmatis* under respiration-inhibitory conditions. Consistent with our assumption, expression of *cydA* in the Δ*devR* (*MSMEG_5244*) mutant of *M. smegmatis* was induced to a similar level as that in the WT strain, when growth of both strains was shifted from aerobic to hypoxic conditions (data not shown). This observation is further supported by the previous report that the *cydAB* operon is not included in 49 genes identified as the DevR regulon (Berney et al., 2014). Our search for the Rex, Fnr, RegB, and ArcB homologs in *M. smegmatis* revealed that their homologous genes are not present in the *M. smegmatis* genome. Our RNA sequencing analysis on Δ*aa*_3_ mutant strain of *M. smegmatis* grown aerobically revealed that 61% of the strongly upregulated DEGs (FC ≥ 4 and *p* < 0.05) in the Δ*aa*_3_ mutant relative to the WT belong to the SigF regulon, indicating that it is the SigF partner switching system that plays a predominant role in strong upregulation of gene expression in *M. smegmatis* under ETC-inhibitory conditions. Since transcription of the *cydAB* operon was shown to be independent of SigF in *M. smegmatis* (Figure 1), there must be (a) regulatory system(s) other than SigF that is (are) responsible for strong upregulation of *cydAB* under respiration-inhibitory conditions. Based on a previous report (Aung et al., 2014) and our results clearly showing that the *cydAB* operon is under the positive regulation of Crp in *M. smegmatis*, we assumed that Crp is involved in upregulation of *cydAB* expression in *M. smegmatis* under respiration-inhibitory conditions. This assumption is supported by our RNA sequencing result that considerable fractions of the genes, which are more than 4-fold upregulated in the Δ*aa*_3_ mutant in a SigF-independent way, were shown to be statistically downregulated in the Δ*crp1* mutant relative to the WT strain.

The Crp proteins are homodimeric global transcription factors that regulate expression of many genes involved in diverse metabolic and cellular processes in prokaryotes, including carbon utilization, respiration, virulence, cell cycle control, reactivation of non-replicating dormant cells, and stress responses, etc., (Utsumi et al., 1989; Rickman et al., 2005; Shimada et al., 2011; Aung et al., 2014; Green et al., 2014; Heroven and Dersch, 2014). The promoter of *cydA* possesses two Crp-binding sites that are centered at positions −65.5 (for CBS2) and −93.5 (for CBS1). Crp was suggested to assist the binding of RNA polymerase to the promoter through its interactions with the C-terminal domain of α-subunit of RNA polymerase, when it serves as an transcriptional activator (Busby and Ebright, 1999; Lawson et al., 2004). Mutagenesis and DNA-binding analyses on CBS1 and CBS2 revealed that mutations in the distal Crp-binding CBS1 more severely affected *cydA* expression than those in CBS2 with a higher binding affinity for Crp1 (Figures 4, 5, 6B), implying that Crp bound at CBS2 might either stabilize the binding of Crp to CBS1 or help the formation of a DNA loop such that Crp bound at CBS1 can participate in recruitment or activation of RNA polymerase. Recently, it was demonstrated that expression of the *cydAB* operon is significantly reduced in a *prrA* null mutant of *M. smegmatis* compared to the WT strain (Maarsingh et al., 2019). However, our EMSA analysis using purified PrrA, which was preincubated with acetyl phosphate for phosphorylation, did not show the binding of PrrA to the upstream region of *cydA*, implying that the PrrBA TCS might indirectly participate in the regulation of the *cydAB* operon (data not shown).

Intracellular concentrations of cAMP in mycobacteria have been found to be significantly higher than those in other bacteria grown under similar conditions (Padh and Venkitasubramanian, 1976; Lee, 1977; Shenoy and Visweswariah, 2006; Bai et al., 2011). Accordingly, it was suggested that mycobacteria have been evolved to possess the Crp proteins that have a low binding affinity for cAMP. Crp1 in *M. smegmatis* and Rv3676 in *M. tuberculosis* are the Crp proteins that have a low binding affinity for cAMP (Stapleton et al., 2010; Sharma et al., 2014). Although purified Rv3676 and Crp1 were shown to bind to their target DNA even in the absence of cAMP in contrast to Crp2 and *E. coli* Crp, their DNA-binding affinity was demonstrated to be enhanced by binding of cAMP (Figures 4A, 5A) (Bai et al., 2005; Rickman et al., 2005; Sharma et al., 2014). Some fast-growing mycobacteria such as *Mycobacterium flavescens, Mycobacterium fortuitum*, and *Mycobacterium phlei* and the pathogenic slow-growing *Mycobacterium avium* contain an additional Crp protein that corresponds to Crp2 in *M. smegmatis* (Sharma et al., 2014). Compared to Crp1, Crp2 of *M. smegmatis* has been reported to have a much higher binding affinity for cAMP (*K*_d_ = ~30 μM for Crp1 and *K*_d_ = ~3 μM for Crp2). It has been also reported that Crp2 does not bind to the target DNA in the absence of cAMP (Sharma et al., 2014). Intracellular levels of cAMP were found to be 3.2-fold increased in *M. smegmatis* by the inactivation of the *aa*_3_ cytochrome *c* oxidase and to rise even more when the aerobically grown WT strain of *M. smegmatis* was subjected to treatment of KCN (Figure 8), which is in line with our previous report that cAMP levels in *M. smegmatis* were increased by ~400- and 5.7-fold under hypoxic and SNP (5 mM)-treated conditions, respectively (Jeon et al., 2014). The genome of *M. smegmatis* contains eight genes encoding adenylyl cyclase (*MSMEG_0228, MSMEG_3578, MSMEG_3780, MSMEG_4279, MSMEG_4477, MSMEG_4924, MSMEG_5018*, and *MSMEG_6154*). Although expression of *MSMEG_3780* and *MSMEG_4279* was observed to increase under both hypoxic and SNP-treated conditions, no detailed study was performed as to which adenylyl cyclase(s) is (are) implicated in an increase in cAMP levels in *M. smegmatis* under respiration-inhibitory conditions (Jeon et al., 2014). Considering both considerably different cAMP-binding affinity of two Crp paralogs and high intracellular levels of cAMP in mycobacteria, it can be assumed that Crp2 with the reportedly 10-fold higher binding affinity for cAMP than Crp1 might exist in a cAMP-bound form, while Crp1 with a low cAMP-binding affinity might be as a cAMP-unbound apo-protein in *M. smegmatis* under normal respiration conditions. If this assumption were true, Crp2 would be inappropriate to serve as a transcription factor that reflects elevating intracellular cAMP levels under respiration-inhibitory conditions to regulate gene expression. Furthermore, both significantly low cellular abundance of Crp2 relative to Crp1 and reduced expression of *crp2* under respiration-inhibitory conditions as a result of the negative regulation of *crp2* by Crp1 (Figures 3, 9) renders Crp2 even more inadequate for a transcription factor that regulates gene expression in response to respiration inhibition. This assumption is consistent with our finding that Crp1, but not Crp2, plays a major role in upregulation of *cydAB* expression under respiration-inhibitory conditions.

In conclusion, we here provided the evidence that Crp1 (MSMEG_6189) of two Crp paralogs in *M. smegmatis* is the major transcription factor that is responsible for upregulation of the *cydAB* operon under ETC-inhibitory conditions. Two Crp-binding sequences were identified upstream of the *cydA* gene, both of which are necessary for induction of *cydAB* expression under ETC-inhibitory conditions. The intracellular level of cAMP in *M. smegmatis* was found to be increased under ETC-inhibitory conditions. The *crp2* gene was found to be negatively regulated by Crp1 and Crp2, which might lead to significantly low cellular abundance of Crp2 relative to Crp1 in *M. smegmatis*.

## Data Availability Statement

The RNA sequencing data have been deposited in NCBI's Gene Expression Omnibus and are accessible through the GEO Series accession number GSE158137.

## Author Contributions

E-MK and J-IO: conception or design of the study, analysis or interpretation of the data, and writing of the manuscript. E-MK: acquisition of the data. All authors contributed to the article and approved the submitted version.

## Conflict of Interest

The authors declare that the research was conducted in the absence of any commercial or financial relationships that could be construed as a potential conflict of interest.
