# Supplementary material for: Induction of the cydAB Operon Encoding the bd Quinol Oxidase Under Respiration-Inhibitory Conditions by the Major cAMP Receptor Protein MSMEG_6189 in Mycobacterium smegmatis
Source: Front Microbiol. 2020 Nov 30;11:608624. doi: 10.3389/fmicb.2020.608624 (PMC7739888; doi:10.3389/fmicb.2020.608624)
Supplement: Supplementary file 1 [file Data_Sheet_1.docx]

**SUPPLEMENTAL MATERIALS**

**Induction of the *cydAB* operon encoding the *bd* quinol oxidase under respiration-inhibitory conditions by the major cAMP receptor protein MSMEG_6189 in *Mycobacterium smegmatis***

Eon-Min Ko and Jeong-Il Oh^*^

*Department of Integrated Biological Science, Pusan National University, 46241 Busan, Korea*

***Correspondence:**

Jeong-Il Oh

Telephone: +82-51-510-2593

Fax: +82-51-514-1778

E-mail: joh@pusan.ac.kr

Running title: Positive regulation of the *cydAB* operon by MSMEG_6189

Keywords: *aa*_3_ cytochrome *c* oxidase, cAMP, Crp, electron transport chain, *Mycobacterium*, regulation of gene expression, respiration

**MATERIALS AND METHODS**

**Construction of mutant strains of *M. smegmatis***

A *crp2* (*MSMEG_0539*) mutantof *M. smegmatis* was constructed by allelic exchange mutagenesis using the temperature-sensitive suicide plasmid pKOTsΔcrp2 containing a temperature-sensitive replication origin as described previously (Jeong et al., 2013). pKOTsΔcrp2 was introduced into *M. smegmatis* by electroporation. Transformants were selected at 30°C (replication-permissive temperature) on 7H9-glucose agar plates containing hygromycin, and the selected transformants were grown in 7H9-glucose liquid medium supplemented with hygromycin for 3 - 5 days at 30°C. Heterogenotes of *M. smegmatis*, which were generated by a single recombination event, were selected for their hygromycin resistance on 7H9-glucose agar plates at 42°C (replication-nonpermissive temperature). The selected heterogenotes were grown on 7H9-glucose medium without antibiotics for 3 - 5 days at 37°C. Isogenic homogenotes were obtained from the heterogenotes after a second recombination by selecting them for sucrose resistance on 7H9-glucose agar plates containing 10% (w/v) sucrose at 37°C. The allelic exchange in the Δ*crp2* mutant was verified by PCR with isolated genomic DNA. The Δ*crp2* mutant is a deletion mutant in which a 250-bp internal fragment is deleted from the *crp2* gene. The deletion results in a frameshift, leading to the complete inactivation of Crp2 including the C-terminal DNA-binding domain.

**Construction of plasmids**

**(i) pKOTsΔcrp2.** To construct pKOTsΔcrp2, PCR was conducted with the F_crp2mut and R_crp2mut primers and the chromosomal DNA of *M. smegmatis* as a template. The amplified 1,086-bp DNA fragment was restricted with HindIII and KpnI and cloned into pBSII KS+ digested with the same enzymes, yielding pBSIIcrp2. The 251-bp DNA fragment within *crp2* was excised from pBSIIcrp2 by restriction with SalI, and the linear plasmid was self-ligated, resulting in pBSIIΔcrp2. Using pBSIIΔcrp2 as a template, an 835-bp DNA fragment was obtained by PCR with *Pfu* DNA polymerase and the primers F_crp2mut and R_crp2mut. The amplified PCR product was restricted with HindIII and cloned into pKOTs digested with HindIII and EcoRV, yielding pKOTsΔcrp2.

**(ii) pNCIIcydA, pNCIISD1, pNCIISD2, pNCIISD3, and pNCIISD4.** pNCIIcydA, pNCIISD1, pNCIISD2, pNCIISD3, and pNCIISD4 are *cydA*::*lacZ* translational fusion plasmids that contain the 5’ portion (54 bp) of *cydA*, as well as 199-, 163-, 143-, 123-, and 83-bp serially deleted DNA sequences upstream of *cydA*, respectively. For the construction of pNCIIcydA, a 253-bp DNA fragment was amplified with the primers F_cydAlacZ and R_cydAlacZ. The PCR product was digested with ClaI and XbaI and cloned into pBSII KS+, resulting in pBSIIcydA. pBSIIcydA was restricted with ClaI and XbaI, and the 253-bp fragment was cloned into pNCII, yielding the pNCIIcydA. To construct pNCIISD1, pNCIISD2, pNCIISD3, and pNCIISD4, the *cydA* upstream regions of the corresponding lengths were amplified by PCR using the forward primers (F_cydASD1, F_cydASD2, F_cydASD3, and F_cydASD4, respectively) and the reverse primer R_cydAlacZ. pBSIIcydA was used as a template for PCR. The PCR products were restricted with ClaI and XbaI and cloned into pNCII, resulting in pNCIISD1, pNCIISD2, pNCIISD3, and pNCIISD4.

**(iii) pNCIIM1, pNCIIM2, and pNCIIM3.** Using pBSIIcydA as a template, PCR-based site-directed mutagenesis was carried out to mutagenize CBS1 and CBS2, resulting in pBSIIM1_1 with mutations in the right half sequence of CBS1 and pBSIIM2_1 with mutations in the left half sequence of CBS2. Using pBSIIM1_1 and pBSIIM2_1, further site-directed mutagenesis was performed to generate pBSIIM1_2 and pBSIIM2_2 with additional mutations in the left half sequence of CBS1 and the right half sequence and CBS2, respectively. For the construction of pBSIIM3_2, the procedure for the construction of pBSIIM1_2 was followed using pBSIIM2_2 as a template. Mutation were verified by DNA sequencing. The 253-bp ClaI-XbaI fragments from pBSIIM1_2, pBSIIM2_2, and pBSIIM3_2 were cloned into pNCII, resulting in the plasmids pNCIIM1, pNCIIM2, and pNCIIM3, respectively.

**(iv) pMV306crp1_2B8 and pMV306crp2_2B8.** To express the C-terminally 2B8 epitope-tagged Crp1 and Crp2 proteins from their own promoters in *M. smegmatis*, the *crp1* and *crp2* genes with the upstream regions encompassing their own promoters and regulatory sequences were cloned into the integration vector pMV306. A 1,006-bp DNA fragment containing the *crp1* gene was amplified using the chromosomal DNA of *M. smegmatis* as a template and the primers F_crp1 and R_crp1 containing the epitope-coding sequence. The PCR product was restricted with KpnI and HindIII, and the restricted DNA fragment was cloned into pMV306, yielding pMV306crp1_2B8. To construct pMV306crp2_2B8, PCR was conducted with the primers F_crp2 and R_crp2 containing the epitope-coding sequence. The amplified 1,006-bp DNA fragment was digested with XbaI and HindIII and cloned into pMV306, resulting in pMV306crp2_2B8.

**(v) pUC19cydAFootR.** The plasmid was used as a template for the generation of fluorescence-labeled DNA fragments for DNase I footprinting analysis. A 220-bp DNA fragment encompassing two Crp-binding sites (CBS1 and CBS2) was amplified by PCR with the primers F_cydAFootR and R_cydAFootR using the chromosomal DNA of *M. smegmatis* as a template. The PCR product was restricted with EcoRI and HindIII and cloned into pUC19, yielding pUC19cydAFootR.


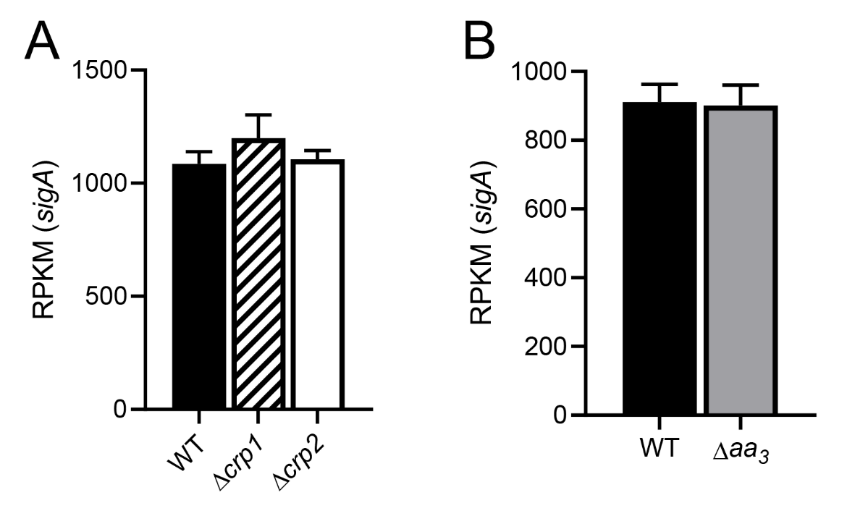


**FIGURE S1. Expression levels of *sigA* in the WT, Δ*crp1*, Δ*crp2*, and Δ*aa_3_* mutant strains of *M. smegmatis*.** Transcript levels of *sigA* were extrapolated from the reads per kilo base pair per million mapped reads (RPKM) values obtained from RNA sequencing analysis on the WT, Δ*crp1*, and Δ*crp2* mutant strains that were grown aerobically to an OD_600_ of 2.0-2.1 (A), as well as the WT and Δ*aa_3_* mutant strains that were grown aerobically to an OD_600_ of 0.45-0.5 (B) (Y. Oh and J. I. Oh, in press).


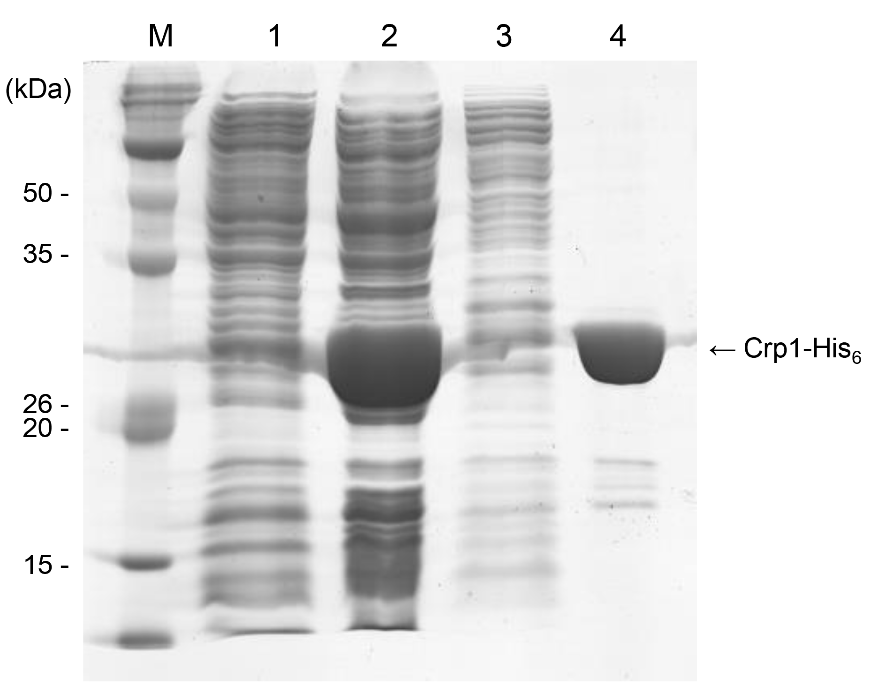


**FIGURE S2. SDS-PAGE analysis of purified Crp1 (MSMEG_6189).** C-terminally His_6_-tagged Crp1 was overexpressed in the *E. coli* BL21 (DE3) strain with pT7-7crp1. The overexpressed Crp1 protein was purified by affinity chromatography using Ni-Sepharose high-performance resin (GE Healthcare, Piscataway, NJ). Lane M, molecular weight marker. Lane 1, lysates of *E. coli* BL21 (DE3) harboring the empty vector pT7-7. Lane 2, lysates of *E. coli* BL21 (DE3) harboring pT7-7crp1. Lane 3, eluents during the washing step with 20 mM Tris-HCl (pH 8.0) containing 60 mM imidazole. Lane 4, purified C-terminally His_6_-tagged Crp1.

**
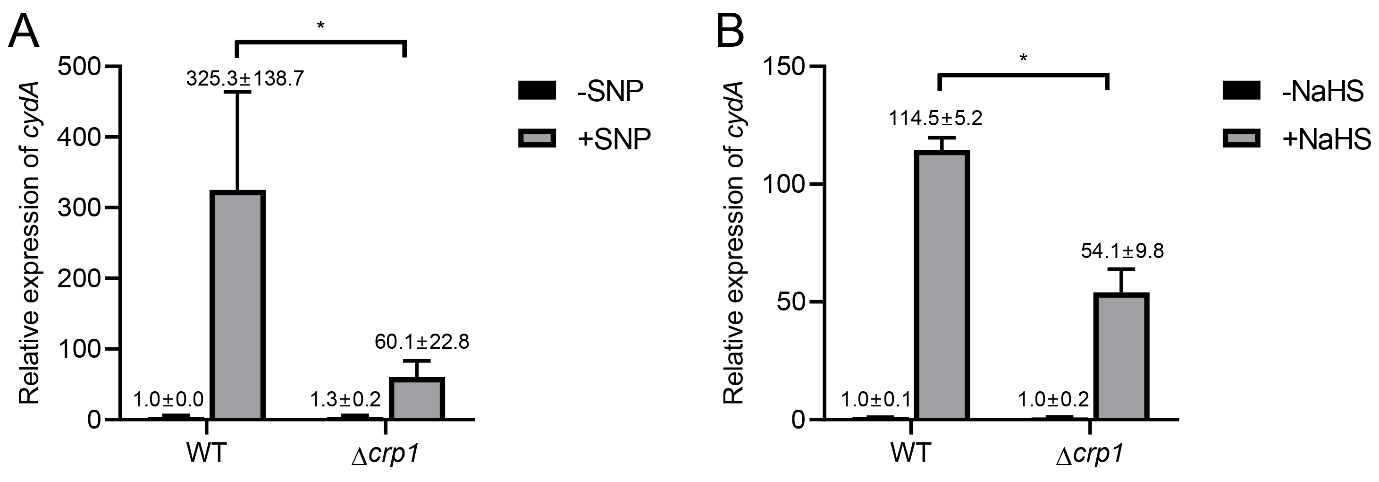
**

**FIGURE S3. Effect of NO and H_2_S treatment on *cydA* expression in the WT and Δ*crp1* mutant strains of *M. smegmatis*.** (A) When the strains were grown aerobically to an OD_600_ of 0.45-0.5, sodium nitroprusside (SNP, NO generator) was added to the cultures with a final concentration of 5 mM and the cultures were further grown under the illumination of light for 30 min (+SNP). As controls, the strains without SNP treatment were included in the experiment (-SNP). (B) The strains were grown aerobically to an OD_600_ of 0.45-0.5 and treated with 200 μM NaHS (H_2_S donor) for 30 min (+NaHS). As controls, the strains without NaHS treatment were included in the experiment (-NaHS). The expression level of *cydA* was quantitatively determined by qRT-PCR and normalized to *sigA* expression. The expression level of the *cydA* gene in the SNP-untreated (A) or NaHS-untreated (B) WT strain is set at 1, and the relative values are expressed for the other strains. All values provide are the averages of the results from three independent experiments. The error bars indicate the standard deviations. Statistical significance was determined by two-tailed Student’s *t* test. *, *p* < 0.05.


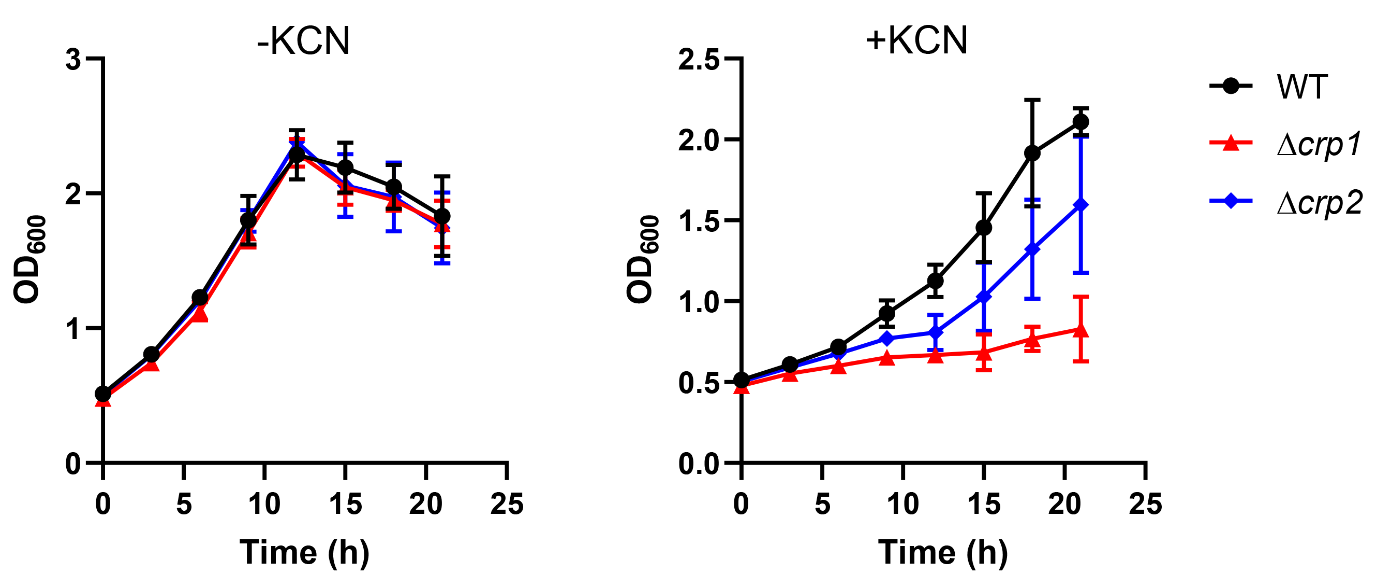


**FIGURE S4. Effect of KCN on aerobic growth of the WT, Δ*crp1*, and Δ*crp2* mutant strains of *M. smegmatis*.** *M. smegmatis* strains were grown aerobically in 7H9-glucose medium to an OD_600_ of 0.5, and the cultures were treated with 100 μM KCN (+KCN). As controls, the *M. smegmatis* strains without treatment of KCN were used in the experiment (-KCN). The cultures were further grown for 21 h, and the growth of the strains was monitored spectrophotometrically at 600 nm at 3-h intervals. All values provided are averages of the results from three independent determinations. The error bars indicate the standard deviations.

**TABLE S1.** Strains and plasmids used in this study

| Strain/plasmid | Relevant phenotype/genotype* | Reference |
| --- | --- | --- |
| Strains |  |  |
| *E.coli* DH5α | ϕ80d*lacZ*ΔM15 Δ*lacU169* *recA1 endA1 hsdR17 supE44 thi1 gyrA96 relA1* | Jessee, 1986 |
| *E.coli* BL21 (DE3) | F^-^, *ompT hsdS_B_* (r_B_^-^, m_B_^-^) *dcm gal* λ (DE3) | Promega |
| *M.* *smegmatis* mc^2^155 | High-transformation-efficiency mutant  of *M. smegmatis* ATCC 607 | Snapper et al., 1990 |
| *M.* *smegmatis* Δ*aa_3_* | *MSMEG_4268* (*ctaC*) deletion mutant derived from *M. smegmatis* mc^2^155 | Jeong et al., 2018 |
| *M.* *smegmatis* Δ*aa_3_*Δ*sigF* | *MSMEG_1804* (*sigF*) deletion mutant derived from *M. smegmatis* mc^2^155 | Oh et al., 2020 |
| *M.* *smegmatis* Δ*crp1* | *MSMEG_6189* (*crp1*) deletion/insertion mutant derived from *M. smegmatis* mc^2^155; Hyg^r^; Previously this mutant was named *crp*. To distinguish *crp1* and *crp2* mutants, we renamed the *crp* mutant to Δ*crp1* | Lee et al., 2014 |
| *M.* *smegmatis* Δ*crp2* | *MSMEG_0539* (*crp2*) deletion mutant derived from *M. smegmatis* mc^2^155 | This study |
| plasmids |  |  |
| pBSII KS+ | Amp^r^; *lacPOZ’* | Stratagene |
| pT7-7 | Amp^r^; T7 promoter, ribosome binding site, and translation start codon overlapping with NdeI site | Tabor and Richardson, 1985 |
| pNCII | Hyg^r^; promoterless *lacZ* | Oh et al., 2020 |
| pUC19 | Amp^r^; *lacPOZ’* | Yanisch-Perron et al., 1985 |
| pKOTs | Hyg^r^; pKO-based vector constructed by inserting the HindIII-KpnI fragment containing pAL500Ts and pUC ori derived from pDE | Jeong et al., 2013 |
| pMV306 | Km^r^; integration vector containing *int* and the *attP* site of mycobacteriophage L5 for integration into the mycobacterial genome | Stover et al., 1991; Brown et al., 2007 |
| pNCIIcydA | pNC2::0.253-kb XbaI-ClaI fragment containing the *cydA* promoter region | This study |
| pNCIISD1 | pNC2::0.217-kb XbaI-ClaI fragment containing the *cydA* promoter region | This study |
| pNCIISD2 | pNC2::0.197-kb XbaI-ClaI fragment containing the *cydA* promoter region | This study |
| pNCIISD3 | pNC2::0.177-kb XbaI-ClaI fragment containing the *cydA* promoter region | This study |
| pNCIISD4 | pNC2::0.137-kb XbaI-ClaI fragment containing the *cydA* promoter region | This study |
| pBSIIcydA | pBSII KS+::0.253-kb XbaI-ClaI fragment containing the *cydA* operon promoter region | This study |
| pBSIIM1_1 | pBSIIcydA with three point mutations (GTG→CCC) in CBS1 | This study |
| pBSIIM1_2 | pBSIIM1_1 with three point mutations (CAC→TTT) in CBS1 | This study |
| pBSIIM2_1 | pBSIIcydA with three point mutations (GTG→CCC) in CBS2 | This study |
| pBSIIM2_2 | pBSIIM2_1 with three point mutations (CAC→TTT) in CBS2 | This study |
| pBSIIM3_1 | pBSIIM1_2 with three point mutations (GTG→CCC) in CBS2 | This study |
| pBSIIM3_2 | pBSIIM3_1 with three point mutations (CAC→TTT) in CBS2 | This study |
| pNCIIM1 | pNC2::0.253-kb XbaI-ClaI fragment from pBSIIM1_2 | This study |
| pNCIIM2 | pNC2::0.253-kb XbaI-ClaI fragment from pBSIIM2_2 | This study |
| pNCIIM3 | pNC2::0.253-kb XbaI-ClaI fragment from pBSIIM3_2 | This study |
| pT7-7crp1 | pT7-7::0.693-kb NdeI-HindIII fragment containing *crp1* (*MSMEG_6189*) with 6 His codons before its stop codon | Bong et al., 2019 |
| pMV306crp | pMV306::1.239-kb ClaI-HindIII fragment containing *crp1* | Lee et al., 2014 |
| pUC19cydAFootR | pUC19::0.219-kb EcoRI-HindIII fragment containing the *cydA* promoter region | This study |
| pKOTsΔcrp2 | pKOTs::0.815-kb EcoRV-HindIII fragment containing Δ*crp2* | This study |
| pMV306crp1_2B8 | pMV306::1.006-kb KpnI-HindIII fragment containing *crp1* | This study |
| pMV306crp2_2B8 | pMV306::1.006-kb XbaI-HindIII fragment containing *crp2* | This study |

*Abbreviations: Amp^r^, ampicillin resistance; Hyg^r^, hygromycin resistance; Km^r^, kanamycin resistance.

**TABLE S2.** Oligonucleotides used in this study

| **Oligonucleotide** | **Nucleotide sequences (5'→3')** | **Purpose** |
| --- | --- | --- |

| F_cydAlacZ | ATATTCTAGATCGGCGTCTTCGAGGTCG | *cydA*::*lacZ* fusion |
| --- | --- | --- |
| R_cydAlacZ | ATATATCGATGTGGTAGACCGTGGTGATTCCG | *cydA*::*lacZ* fusion |
| F_crp2mut | ATATGGTACCGACGAAGTGCTGGCGC | Δ*crp2* construction |
| R_crp2mut | ATATAAGCTTGCTTGAGGGTGAGGCCACAAC | Δ*crp2* construction |
| F_cydASD1 | ATATTCTAGAGCAGCGGGGTCAGGAAGC | *cydA*::*lacZ* fusion |
| F_cydASD2 | ATATTCTAGACTCACCATCGGCGCCTCC | *cydA*::*lacZ* fusion |
| F_cydASD3 | ATATTCTAGAGGCGCCGCCTTGTCGACC | *cydA*::*lacZ* fusion |
| F_cydASD4 | ATATTCTAGACCTGCGTCGATCTCCTGGC | *cydA*::*lacZ* fusion |
| F_cydAM1_1 | CTTGTCGACCGGCTTTTCATCGGCGCCTCCC | Site-directed mutagenesis (CBS1) |
| R_cydAM1_1 | GGGAGGCGCCGATGAAAAGCCGGTCGACAAG | Site-directed mutagenesis (CBS1) |
| F_cydAM1_2 | CTCGGCGCCGCCTTCCCGACCGGCTTTTCAT | Site-directed mutagenesis (CBS1) |
| R_cydAM1_2 | ATGAAAAGCCGGTCGGGAAGGCGGCGCCGAG | Site-directed mutagenesis (CBS1) |
| F_cydAM2_1 | CATCGGCGCCTCCCCCCAGCTAACCCACCGC | Site-directed mutagenesis (CBS2) |
| R_cydAM2_1 | GCGGTGGGTTAGCTGGGGGGAGGCGCCGATG | Site-directed mutagenesis (CBS2) |
| F_cydAM2_2 | CCCCCCAGCTAACCTTTCGCGCTACCTGCGT | Site-directed mutagenesis (CBS2) |
| R_cydAM2_2 | ACGCAGGTAGCGCGAAAGGTTAGCTGGGGGG | Site-directed mutagenesis (CBS2) |
| F_crp1 | ATATGGTACCCCCGCGAGCAGGCACCA C | Δ*crp1* complementation |
| R_crp1 | ATATAAGCTTTCAGGGGGGGAAGGCGGGCAGCGGGTCGCGGCGGGCGCGGCGGGCCAG | Δ*crp1* complementation |
| F_crp2 | ATATTCTAGAGTCGTAGAGCAACGGCAGCG | Δ*crp2* complementation |
| R_crp2 | ATATAAGCTTTCAGGGGGGGAAGGCGGGCAGGGGGTCGCGGTTCGCGCGCCGCGCGAG | Δ*crp2* complementation |
| F_cydAFootR | ATATAAGCTTCGTCCTCGGCGTCTTCG | DNase I footprinting |
| R_cydAFootR | ATATGAATTCGTCCAGAGCGTCCATCTGACC | DNase I footprinting |
| F_TAMRA_pUC19 | TAMRA-GTTTTCCCAGTCACGACGTTGTA | DNase I footprinting |
| F_cydAEMSA | GCAGCGGGGTCAGGAAGC | EMSA (specific DNA) |
| R_cydAEMSA | GCCAGGAGATCGACGCAGG | EMSA (specific DNA) |
| F_80_EMSA | CATCCCCCTTTCGCCAGC | EMSA (control DNA) |
| R_80_EMSA | CCATTCAGGCTGCGCAAC | EMSA (control DNA) |
| F_sigA_RT | CTTGAGGTGACCGACGATCT | qRT-PCR |
| R_sigA_RT | AGCTTCTTCTTCCTCGTCCT | qRT-PCR |
| F_cydA_RT | CGGTGGCAGTTCGGAATCAC | qRT-PCR |
| R_cydA_RT | CAGAAAAAGTTTGCCGAAGAAACG | qRT-PCR |
| F_3680_RT | GTTTGCCGCAGCGCTCGC | qRT-PCR |
| R_3680_RT | CCCCACCCGGTTCACCAC | qRT-PCR |
| F_crp2_RT | GGCATCTTCCAGGGGGTGC | qRT-PCR |
| R_crp2_RT | CGGCCGTCCACCGACTTG | qRT-PCR |

**TABLE S3.** Summarized statistics of RNA sequencing alignment

|  | **WT_1** | **WT_2** | **WT_3** | **Δ*crp1*_1** | **Δ*crp1*_2** | **Δ*crp1*_3** |
| --- | --- | --- | --- | --- | --- | --- |
| Read length (bp) | 101 | 101 | 101 | 101 | 101 | 101 |
| Total reads | 44,006,244 | 58,518,714 | 42,824,056 | 44,214,864 | 48,894,924 | 39,772,582 |
| No. of reads after trimming | 42,632,572 | 56,994,762 | 40,838,124 | 42,556,276 | 47,698,382 | 38,517,206 |
| No. of processed reads^a^ | 21,316,286 | 28,497,381 | 20,419,062 | 21,278,138 | 23,849,191 | 19,258,603 |
| No. of mapped reads | 11,876,302 | 19,947,198 | 6,274,069 | 6,827,931 | 12,209,518 | 6,256,176 |
| No. of failed-to-align reads^b^ | 4,045,381 | 4,090,121 | 3,376,054 | 3,501,038 | 4,944,196 | 3,536,126 |
| No. of suppressed reads by multiple mapping^c^ | 5,394,603 | 4,460,062 | 10,768,939 | 10,949,169 | 6,695,477 | 9,466,301 |

^a^The number of the processed reads indicates the number of the reads that remained after preprocessing of the total reads, e.g., removal of adapter sequences and artifacts such as contaminant DNA and PCR duplicates, etc. The processed reads were used for mapping to the reference genome using the program Bowtie v1.1.2.

^b^The number of failed-to-align reads indicates the number of the reads that were not mapped to the reference genome due to the sequence mismatch.

^c^The number of suppressed reads by multiple mapping indicates the number of the reads that were not mapped to the reference genome due to multiple mapping of the reads to two or more genes.

**TABLE S4.** The genes that are upregulated in the Δ*aa_3_* mutant of *M. smegmatis* relative to the WT strain in a SigF-independent way and belong to the Crp1 regulon

|  | |  | **Δ*aa_3_*/WT** | | **Δ*crp1*/WT** | |
| --- | --- | --- | --- | --- | --- | --- |
| **Locus tag** | **Description** | | **log_2_FC** | ***p*-value** | **log_2_FC** | ***p*-value** |
| MSMEG_0265 | UdgX family uracil-DNA binding protein | | 3.2 | 0.024 | -1.0 | 0.000 |
| MSMEG_0430 | | ISL3-like element ISMsm4 family transposase | 2.8 | 0.023 | -0.9 | 0.005 |
| MSMEG_0450 | | Hypothetical protein | 2.9 | 0.029 | -0.8 | 0.000 |
| MSMEG_0637 | | FAD-dependent oxidoreductase | 2.7 | 0.046 | -1.2 | 0.000 |
| MSMEG_0651 | | Hypothetical protein | 2.7 | 0.000 | -0.8 | 0.001 |
| MSMEG_1097 | | Glycosyltransferase | 3.0 | 0.044 | -1.0 | 0.000 |
| MSMEG_1315 | | Mechanosensitive ion channel family protein | 2.3 | 0.046 | -0.7 | 0.000 |
| MSMEG_1605 | | Phosphate signaling complex protein PhoU | 2.8 | 0.028 | -0.9 | 0.000 |
| MSMEG_1773 | | Iron-containing redox enzyme family protein | 2.7 | 0.035 | -1.2 | 0.000 |
| MSMEG_1970 | | GAF domain-containing protein | 3.1 | 0.007 | -0.8 | 0.000 |
| MSMEG_3232 | | Cytochrome *d* ubiquinol oxidase subunit II | 3.0 | 0.000 | -0.7 | 0.000 |
| MSMEG_3233 | | Cytochrome ubiquinol oxidase subunit I | 2.8 | 0.000 | -0.9 | 0.000 |
| MSMEG_3418 | | SRPBCC family protein | 2.5 | 0.042 | -1.5 | 0.000 |
| MSMEG_3543 | | Protein disulfide oxidoreductase | 2.6 | 0.028 | -0.8 | 0.000 |
| MSMEG_3680 | | Hypothetical protein | 2.1 | 0.004 | -3.0 | 0.000 |
| MSMEG_3865 | | Hypothetical protein | 2.5 | 0.006 | -2.1 | 0.000 |
| MSMEG_4195 | | Hypothetical protein | 2.2 | 0.026 | -0.8 | 0.000 |
| MSMEG_4465 | | Cutinase family protein | 2.8 | 0.002 | -3.2 | 0.000 |
| MSMEG_4618 | | Cysteine hydrolase | 2.5 | 0.030 | -0.6 | 0.000 |
| MSMEG_5559 | | Sugar porter family MFS transporter | 2.3 | 0.029 | -1.0 | 0.000 |
| MSMEG_5722 | | Hypothetical protein | 3.4 | 0.015 | -0.6 | 0.000 |
| MSMEG_6233 | | Hypothetical protein | 2.5 | 0.038 | -1.0 | 0.002 |
| MSMEG_6345 | | Hypothetical protein | 2.1 | 0.009 | -0.6 | 0.000 |
| MSMEG_6498 | | Hypothetical protein | 2.1 | 0.000 | -2.7 | 0.000 |
| MSMEG_6610 | | DUF58 domain-containing protein | 2.6 | 0.049 | -1.4 | 0.000 |

**REFERENCES**

Bong, H. J., Ko, E. M., Song, S. Y., Ko, I. J., and Oh, J. I. (2019). Tripartite Regulation of the *glpFKD* Operon Involved in Glycerol Catabolism by GylR, Crp, and SigF in *Mycobacterium smegmatis*. *J. Bacteriol.* 201**,** e00511-00519. doi: 10.1128/JB.00511-19.

Brown, A. K., Bhatt, A., Singh, A., Saparia, E., Evans, A. F., and Besra, G. S. (2007). Identification of the dehydratase component of the mycobacterial mycolic acid-synthesizing fatty acid synthase-II complex. *Microbiology* 153**,** 4166-4173. doi: 10.1099/mic.0.2007/012419-0.

Jeong, J. A., Lee, H. N., Ko, I. J., and Oh, J. I. (2013). Development of new vector systems as genetic tools applicable to mycobacteria. *J. Life Science.* 23**,** 290-298. doi: 10.5352/JLS.2013.23.2.290.

Jeong, J. A., Park, S. W., Yoon, D., Kim, S., Kang, H. Y., and Oh, J. I. (2018). Roles of Alanine Dehydrogenase and Induction of Its Gene in *Mycobacterium smegmatis* under Respiration-Inhibitory Conditions. *J. Bacteriol.* 200**,** e00152-00118. doi: 10.1128/JB.00152-18.

Jessee, J. (1986). New subcloning efficiency competent cells: >1x10^6^ transformants/μg. *Focus* 8**,** 9-10.

Lee, H. N., Lee, N. O., Han, S. J., Ko, I. J., and Oh, J. I. (2014). Regulation of the *ahpC* gene encoding alkyl hydroperoxide reductase in *Mycobacterium smegmatis*. *PLoS One* 9**,** e111680. doi: 10.1371/journal.pone.0111680.

Oh, Y., Song, S. Y., Kim, H. J., Han, G., Hwang, J., Kang, H. Y., et al. (2020). The Partner Switching System of the SigF Sigma Factor in *Mycobacterium smegmatis* and Induction of the SigF Regulon Under Respiration-Inhibitory Conditions. *Front. Microbiol.* 11**,** 588487. doi: 10.3389/fmicb.2020.588487.

Snapper, S. B., Melton, R. E., Mustafa, S., Kieser, T., and Jacobs, W. R. Jr. (1990). Isolation and characterization of efficient plasmid transformation mutants of *Mycobacterium smegmatis*. *Mol. Microbiol.* 4**,** 1911-1919. doi: 10.1111/j.1365-2958.1990.tb02040.x.

Stover, C. K., de la Cruz, V. F., Fuerst, T. R., Burlein, J. E., Benson, L. A., Bennett, L. T., et al. (1991). New use of BCG for recombinant vaccines. *Nature* 351**,** 456-460. doi: 10.1038/351456a0.

Tabor, S., and Richardson, C. C. (1985). A bacteriophage T7 RNA polymerase/promoter system for controlled exclusive expression of specific genes. *Proc. Natl. Acad. Sci. U. S. A.* 82**,** 1074-1078. doi: 10.1073/pnas.82.4.1074.

Yanisch-Perron, C., Vieira, J., and Messing, J. (1985). Improved M13 phage cloning vectors and host strains: nucleotide sequences of the M13mp18 and pUC19 vectors. *Gene* 33**,** 103-119. doi: 10.1016/0378-1119(85)90120-9.
